# BRAF inhibitors suppress apoptosis through off-target inhibition of JNK signaling

**DOI:** 10.7554/eLife.00969

**Published:** 2013-11-05

**Authors:** Harina Vin, Sandra S Ojeda, Grace Ching, Marco L Leung, Vida Chitsazzadeh, David W Dwyer, Charles H Adelmann, Monica Restrepo, Kristen N Richards, Larissa R Stewart, Lili Du, Scarlett B Ferguson, Deepavali Chakravarti, Karin Ehrenreiter, Manuela Baccarini, Rosamaria Ruggieri, Jonathan L Curry, Kevin B Kim, Ana M Ciurea, Madeleine Duvic, Victor G Prieto, Stephen E Ullrich, Kevin N Dalby, Elsa R Flores, Kenneth Y Tsai

**Affiliations:** Department of Immunology, University of Texas MD Anderson Cancer Center, Houston, United States; Graduate School of Biomedical Sciences at Houston, University of Texas, Houston, United States; Department of Dermatology, University of Texas MD Anderson Cancer Center, Houston, United States; Division of Medicinal Chemistry, College of Pharmacy, University of Texas, Austin, United States; Department of Biochemistry and Molecular Biology, University of Texas MD Anderson Cancer Center, Houston, United States; Max F Perutz Laboratories, Vienna, Austria; Center for Oncology and Cell Biology, Feinstein Institute for Medical Research, Manhasset, United States; Department of Pathology, University of Texas MD Anderson Cancer Center, Houston, United States; Department of Melanoma Medical Oncology, University of Texas MD Anderson Cancer Center, Houston, United States; University of Massachusetts Medical School, United States

**Keywords:** protein kinase, cancer, apoptosis, melanoma, squamous cell carcinoma, targeted therapy, Human, Mouse

## Abstract

Vemurafenib and dabrafenib selectively inhibit the v-Raf murine sarcoma viral oncogene homolog B1 (BRAF) kinase, resulting in high response rates and increased survival in melanoma. Approximately 22% of individuals treated with vemurafenib develop cutaneous squamous cell carcinoma (cSCC) during therapy. The prevailing explanation for this is drug-induced paradoxical ERK activation, resulting in hyperproliferation. Here we show an unexpected and novel effect of vemurafenib/PLX4720 in suppressing apoptosis through the inhibition of multiple off-target kinases upstream of c-Jun N-terminal kinase (JNK), principally ZAK. JNK signaling is suppressed in multiple contexts, including in cSCC of vemurafenib-treated patients, as well as in mice. Expression of a mutant ZAK that cannot be inhibited reverses the suppression of JNK activation and apoptosis. Our results implicate suppression of JNK-dependent apoptosis as a significant, independent mechanism that cooperates with paradoxical ERK activation to induce cSCC, suggesting broad implications for understanding toxicities associated with BRAF inhibitors and for their use in combination therapies.

**DOI:** http://dx.doi.org/10.7554/eLife.00969.001

## eLife digest

Over 50% of melanomas, a highly lethal form of skin cancer, carry mutations in a gene called BRAF. The BRAF gene encodes an enzyme that helps to regulate the proliferation of cells, but mutations in this gene lead to the excessive proliferation that is seen in cancer. Clinical trials have shown that a drug called vemurafenib can be used to treat patients who carry the mutated BRAF genes and go on to develop melanoma, but around one fifth of these patients developed another type of skin cancer called cSCC (cutaneous squamous cell carcinoma).

The cSCC tumors often develop in areas where the sun has damaged the patient’s skin, and it is thought that their growth is then accelerated by vemurafenib activating another enzyme, ERK, which causes the excessive proliferation of skin cells. Vin et al. have now found that vemurafenib might also cause cSCC tumors by blocking another signaling pathway. The experiments were performed in human cells and also in mice, and the results were then verified in human cSCC samples.

Cells that are exposed to UV radiation usually die, but when treated with vemurafenib, some 70% of the cells that would have died instead survived. The stress from the UV radiation activates the JNK signaling pathway, which causes the irradiated cells to die. However, Vin et al. found that cSCC cells had very low levels of JNK signaling because treatment with vemurafenib had the unintended effect of inhibiting three enzymes that are needed to fully activate the JNK signaling pathway.

Vin et al. estimate that suppression of JNK signaling and cell death is responsible for about 17.6 to 40% of the effect on cSCC growth seen in melanoma patients, with activation of the ERK pathway accounting for the rest. These unexpected findings suggest that combining vemurafenib treatment with radiation or chemotherapy should be done with caution as these effects could affect their efficacy. It also suggests that future drugs should be designed in a way that avoids these types of effects by making sure they do not inhibit important ‘off-target’ enzymes.

**DOI:** http://dx.doi.org/10.7554/eLife.00969.002

## Introduction

BRAF inhibitors (BRAFi) have revolutionized the treatment of melanoma (Flaherty et al., 2010; Chapman et al., 2011; Sosman et al., 2012; Falchook et al., 2012; Hauschild et al., 2012; Long et al., 2012). Their clinical use is associated with the development of keratinocytic tumors including cSCC (Flaherty et al., 2010; Chapman et al., 2011; Sosman et al., 2012; Hauschild et al., 2012; Falchook et al., 2012; Long et al., 2012). Mechanistic studies of this have centered on paradoxical ERK activation, which is most evident in *BRAF*-wild-type, *RAS*-mutant cells, as the primary mechanism (Karreth et al., 2009; Halaban et al., 2010; Hatzivassiliou et al., 2010; Heidorn et al., 2010; Poulikakos et al., 2010). This is supported by the findings that *RAS* mutations are significantly enriched in cSCC arising in patients treated with vemurafenib relative to sporadic cSCC (Oberholzer et al., 2011; Su et al., 2012), and by the low rate of cSCC in patients treated with combined BRAFi and MEK inhibitor (MEKi) (Flaherty et al., 2012). In one model, drug binding relieves the autoinhibition of BRAF whereupon it is recruited to the membrane by activated RAS and dimerizes with CRAF, driving MEK-dependent ERK activation (Heidorn et al., 2010). Other studies show ERK hyperactivation resulting from drug-induced CRAF transactivation (Hatzivassiliou et al., 2010; Poulikakos et al., 2010) and modulation of RAS spatiotemporal dynamics (Cho et al., 2012). Inhibitor-induced KSR1-BRAF dimers modulate the activity of ERK (McKay et al., 2011) and also affect MEK signaling by activating KSR1 kinase activity (Brennan et al., 2011; Hu et al., 2011). These models all highlight the importance of CRAF in driving MEK-dependent hyperactivation of ERK.

Because of the rapid development of these cSCC on BRAFi therapy and the enrichment for *RAS* mutations, pre-existing genetic lesions are likely present prior to therapy, which are then ‘unmasked’ following initiation of BRAFi therapy. The fact that many arise in sun-damaged skin suggests that prior chronic UV exposure is an important predisposing event (Su et al., 2012).

We instead hypothesized that vemurafenib and PLX4720 could also affect the susceptibility of cells to apoptosis and in so doing, contribute to the acceleration of tumor development. We studied the acute ultraviolet radiation (UVR) response because this is the most important environmental risk factor in the development of skin cancer and because many BRAFi-induced cSCC arise in sun-damaged areas (Su et al., 2012). PLX4720 and vemurafenib share structural features (Tsai et al., 2008; Bollag et al., 2010) and have similar activities, as is the case in our studies.

## Results

### BRAFi suppress stress-induced, JNK-dependent apoptosis

**Figure 1.**
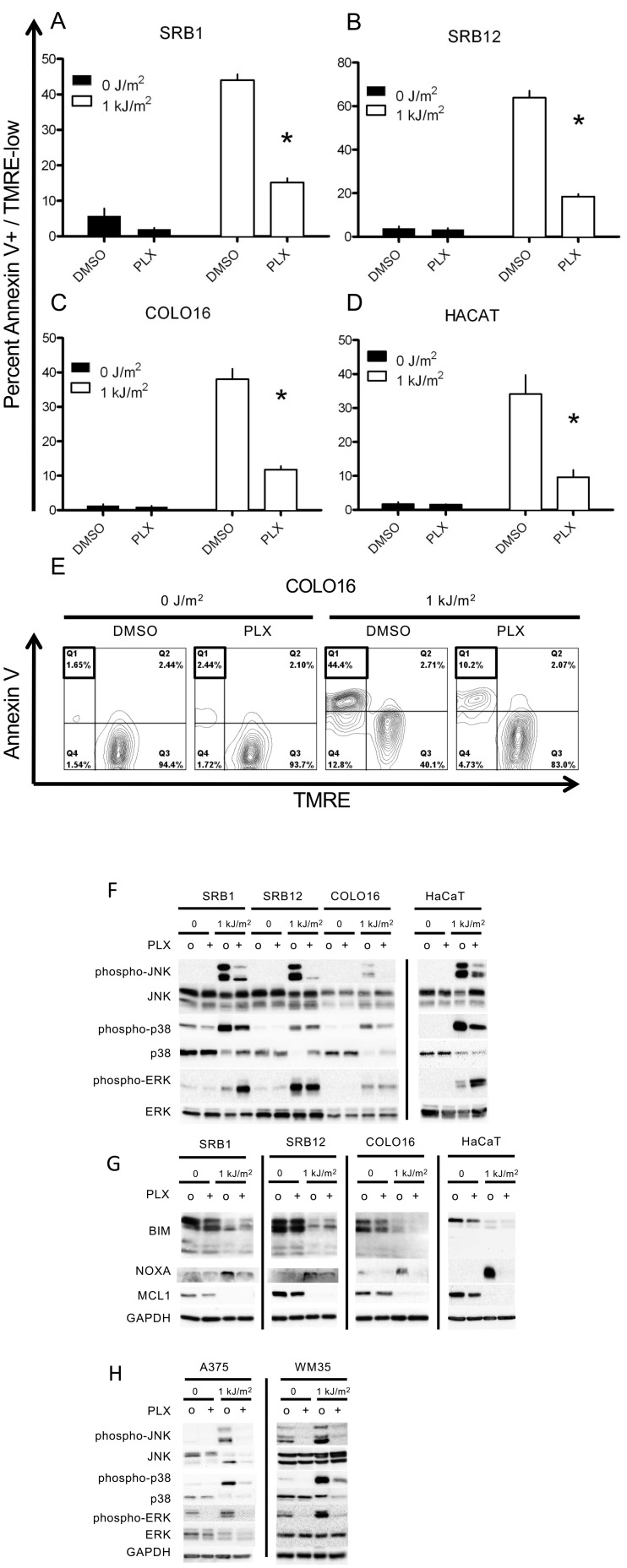
PLX4720 suppresses UV-induced apoptosis. The cSCC and HaCaT cell lines were either unirradiated or irradiated with 1 kJ/m^2^ of UVB in the absence (‘o’, 1:2000 DMSO) or presence (‘+’) of 1 μM PLX4720 and isolated for FACS analysis and protein extracts 24 hr later. (**A**) SRB1, (**B**) SRB12, (**C**) COLO16, and (**D**) HaCaT cells show at least 70% suppression of apoptosis in the presence of PLX4720 as measured by FACS for Annexin V+, TMRE-low cells (n = 6 for each cell line, ‘*’ denotes statistical significance at p<0.05). (**E**) A representative FACS plot for COLO16 is shown. Annexin V+, TMRE-low cells are contained in the upper left quadrant (boxed), which was significantly populated in UV-irradiated cells, but not in the absence of UV, or in the presence of PLX4720. (**F**) Western blots probed for the MAP kinases demonstrated strong phospho-JNK and phospho-p38 induction following irradiation and significant suppression by PLX4720. Phospho-ERK was induced following irradiation, and at 24 hr, paradoxical hyperactivation in the presence of PLX4720 was observed in SRB1 and HaCaT cells. (**G**) Western blots showed that BIM was not upregulated in these *BRAF*-wild-type cells, consistent with intact ERK signaling. MCL1 was downregulated by irradiation and not modulated by PLX4720, whereas NOXA expression was strongly induced in irradiated cells and suppressed by PLX4720. (**H**) Western blots of *BRAF*^*V600E*^ melanoma cell lines, A375 and WM35, demonstrated suppression of UV-mediated induction of phospho-JNK and phospho-p38 by PLX4720 at 24 hr. As expected, phospho-ERK is shut down in PLX4720-treated cells. **DOI:** http://dx.doi.org/10.7554/eLife.00969.003

We performed our initial studies using cSCC (SRB1, SRB12, COLO16) and keratinocyte (HaCaT) cell lines. Cells treated with 1 kJ/m^2^ of UVB (FS40 lamp) undergo apoptosis within 24 hr (Figure 1A–D). Surprisingly, this apoptosis was suppressed by at least 70% in cells concomitantly treated with 1 μM PLX4720 (Figure 1A–D) compared to control DMSO-treated cells as measured by FACS for Annexin V+; TMRE (tetramethylrhodamine)-low cells (Figure 1E, Figure 1—figure supplement 1A–C). Similar results were obtained using doxorubicin as the inducer of apoptosis, and similar suppression of apoptosis was obtained using 1 μM PLX4720 in all cells (Figure 1—figure supplement 2A,B). Importantly, these cells have no oncogenic *RAS* or *BRAF* mutations (Table 1), and PLX4720 conferred no significant proliferative advantage to the tested cells (Figure 1—figure supplement 3) even when used at concentrations that inhibit the proliferation of *BRAF*^*V600E*^ melanoma cell lines (Tsai et al., 2008).

**Figure 1—figure supplement 1.**
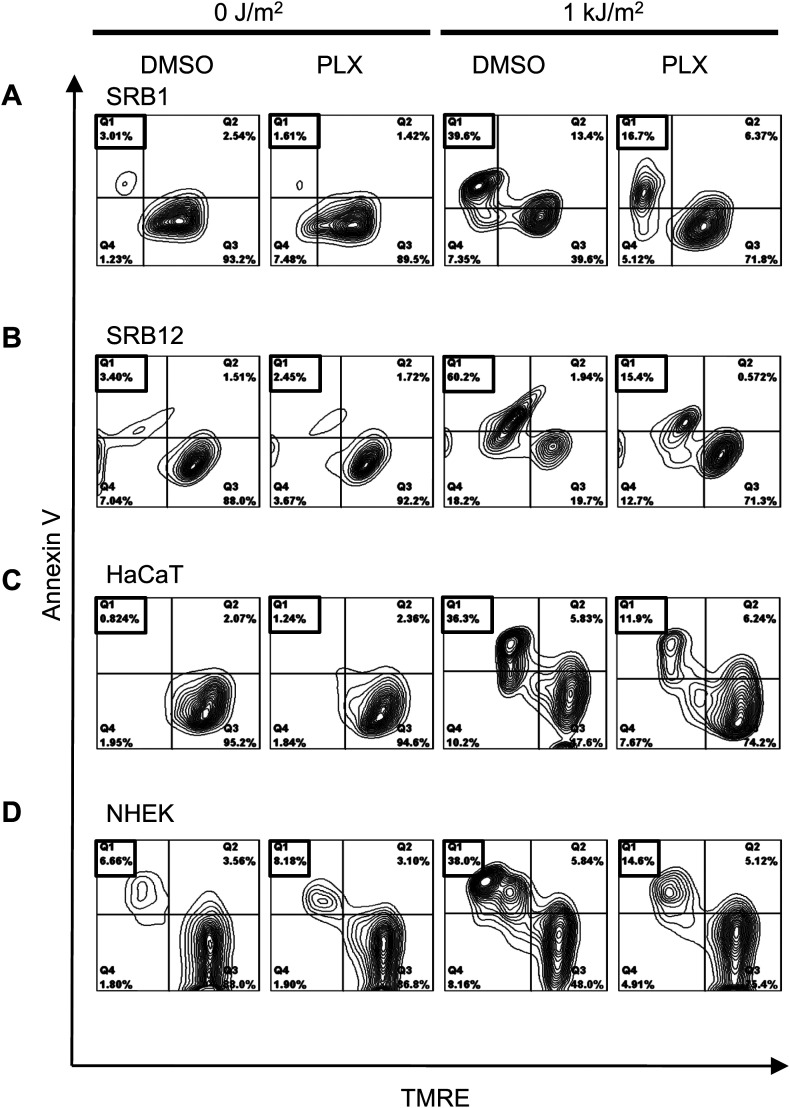
PLX4720 potently suppresses apoptosis in cSCC, HaCaT cell lines, and NHEK cells. Representative FACS plots of Annexin V vs TMRE in SRB1 (**A**), SRB12 (**B**), HaCaT (**C**), and NHEK (**D**) cells demonstrated low levels of apoptosis (Annexin V+, TMRE-low in quadrant 1) in unirradiated cells in the presence and absence of 1 μM PLX4720. Significant levels of apoptosis were seen in all control-treated irradiated cells, which were significantly suppressed in the presence of PLX4720, by at least 70% in all cells tested. **DOI:** http://dx.doi.org/10.7554/eLife.00969.004

**Figure 1—figure supplement 2.**
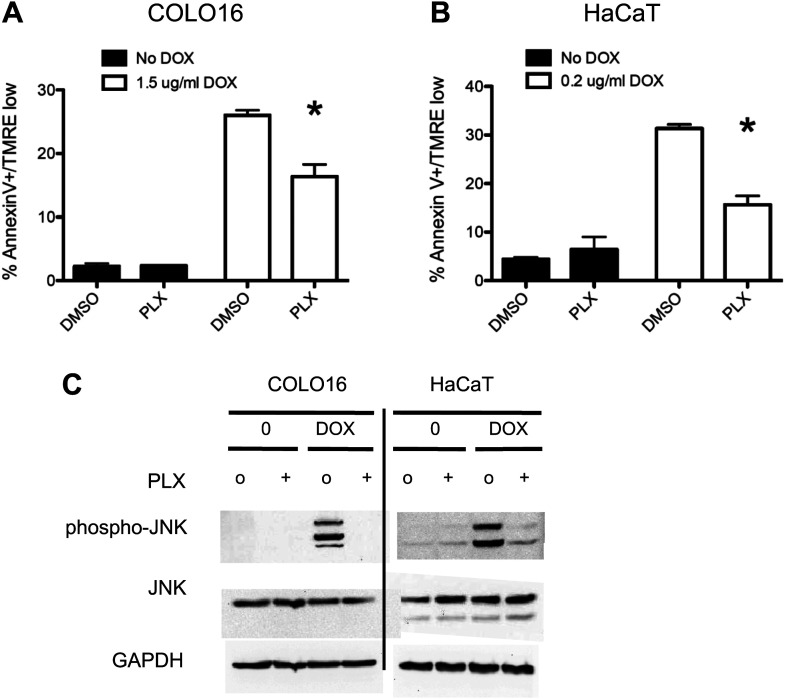
PLX4720 suppresses doxorubicin-induced JNK activation and apoptosis in cSCC and HaCaT cell lines. COLO16 and HaCaT cell lines were either treated with doxorubicin or PBS and lysed 24 hr later in the absence (‘o’, 1:2000 DMSO) or presence (“+”) of 1 μM PLX4720. (**A**) COLO16 and (**B**) HaCaT cells showed significant decrease in apoptosis measured by FACS for Annexin V+, TMRE-low cells (n = 3 for each cell line, ‘*’ denotes statistical significance at p<0.05). (**C**) Western blots were probed for phospho-JNK and total JNK, showing a potent activation of JNK by doxorubicin that is significantly suppressed by PLX4720. **DOI:** http://dx.doi.org/10.7554/eLife.00969.005

**Figure 1—figure supplement 3.**
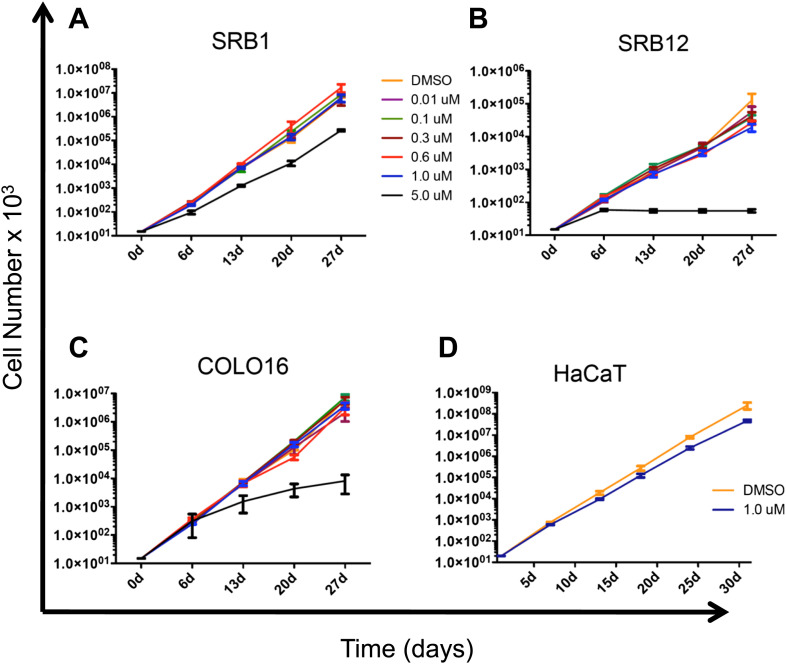
PLX4720 does not confer a proliferative advantage to cSCC and HaCaT cell lines. (**A**) SRB1, (**B**) SRB12, (**C**) COLO16, and (**D**) HaCaT cells were treated with DMSO (1:2000) or the indicated concentrations of PLX4720 for at least 28 days during which cells were serially passaged and counted. Over that time frame there was a slight decrement in the proliferation of SRB12 and HaCaT cells in the presence of 1 μM PLX4720. All cells treated at 5 μM PLX4720 exhibited decreased proliferation. **DOI:** http://dx.doi.org/10.7554/eLife.00969.006

**Table 1.** Lack of *BRAF* and *RAS* mutations in cSCC and HaCaT cell lines **DOI:** http://dx.doi.org/10.7554/eLife.00969.007

ALK_F1174LIV_T3520CAG	ALK_F1245C_T3734G
ALK_F1245VI_T3733GA	BRAF_G464EVA_G1391ATC
BRAF_G466R_G1396CA	BRAF_K601E_A1801G
BRAF_V600EAG_T1799ACG_F	CTNNB1_S45APT_T133GCA
CTNNB1_T41APS_A121GCS	EGFR_Y813C_A2438G
GNAS_R201SC_C601AT	KRAS_G12SRC_G34ACT
KRAS_Q61EKX_C181GAT	MET_H1112_A3335GT
MET_Y1248HD_T3742CG	PIK3CA_A1046V_C3137T
PIK3CA_C420R_T1258C	PIK3CA_E110K_G328A
PIK3CA_E418K_G1252A	PIK3CA_F909L_C2727G
PIK3CA_H1047RL_A3140 GT	PIK3CA_H701P_A2102C
PIK3CA_N345K_T1035A	PIK3CA_Q060K_C178A
PIK3CA_R088Q_G263A	PIK3CA_S405F_C1214T
TNK2_R99Q_G296A	BRAF_G466EVA_G1397ATC
BRAF_V600LM_G1798 TA	CTNNB1_S37APT_T109GCA
CTNNB1_S45CFY_C134GTA	EGFR_G719_G2155TA
EGFR_L858R_T2573G	EGFR_T790M_C2369T
EPHA3_K761N_G2283	FGFR2_S252W_C755G
FOXL2_C134W_C402G	KIT_K642E_A1924G
KIT_R634W_C1900T	KIT_V560D_T1679A
KIT_V825A_T2474C	KIT_Y553N_T1657A
KRAS_G12DAV_G35ACT	MET_N375S_A1124G
NRAS_G12SRC_G34ACT	PIK3CA_E453K_G1357A
PIK3CA_E545AGV_A1634CGT	PIK3CA_H1047RL_A3140 GT..1.
PIK3CA_K111N_G333C	PIK3CA_M1043V_A3127G
PIK3CA_P539R_C1616G	BRAF_E586K_G1756A
BRAF_G469EVA_G1406ATC	CTNNB1_S33APT_T97GCA
CTNNB1_S37CFY_C110GTA	EGFR_L861_T2582AG
EGFR_T854I_C2561T	FGFR2_N549KK_T1647GA
FRAP_R2505P_G7514C	FRAP_S2215Y_C6644T
IDH2_R172MK_G515 TA	JAK2_V617F_G1849T
KIT_L576P_T1727C	KIT_N566D_A1696 G
KRAS_A146PT_G436CA	NRAS_G12DAV_G35ACT
KRAS_Q61HHE_A183CTG	KRAS_G13SRC_G37ACT
NRAS_G13DAV_G38ACT	NRAS_Q61HHQ_A183TCG
PDGFRA_N659Y_A1975T	PDGFRA_V561D_T1682A
PIK3CA_E545KQ_G1633AC	PIK3CA_H1047Y_C3139T
PIK3CA_Q546EK_C1636 GA	PIK3CA_Y1021HN_T3061CA
RET_M918T_T2753C	AKT1_G173R_G517C
AKT2_E17K_G49A	BRAF_G469R_G1405CA
BRAF_L597R_T1790G	BRAF_V600_G1800
CTNNB1_G34EVA_G101ATC	EGFR_S720P_T2158C
GNA11_Q209LP_A626 TC	IDH1_R132CGS_C394TGA
IDH2_R140LQ_G419 TA	IDH2_R140W_C418T
IDH2_R172S_G516T	KIT_D816HNY_G2446CAT
KIT_V559ADG_T1676CAG	KRAS_G10R_G28A
KRAS_Q61LPR_A182TCG	MET_H1112Y_C3334T
MET_M1268T_T3803C	MET_T1010I_C3029T
NRAS_A146T_G436A	NRAS_Q61EKX_C181GAT
PDGFRA_D842V_A2525T	PDGFRA_D842_G2524TA
PDGFRA_N659K_C1977A	PIK3CA_E542KQ_G1624AC
PIK3CA_G1049R_G3145C	PIK3CA_M1043I_G3129ATC
PIK3R1_D560Y_G1678T	PRKAG2_N488I_A1463T
AKT2_G175R_G523C	AKT3_G171R_G511A
ALK_F1174L_C3522AG	ALK_I1171N_T3512A
ALK_R1275QL_G3824AT	BRAF_D594GV_A1781 GT
CTNNB1_D32HNY_G94CAT	FBWX7_R465C_C1393T
FBWX7_R479QL_G1436AT	FBWX7_R505HLP_G1514ATC
FGFR3_G370C_G1108T	GNAQ_Q209H_A627T
IDH2_R140W_C419T	IDH2_R172GW_A514 GT
KIT_N822KNK_T2466GCA	KRAS_G13DAV_G38ACT
PDPK1_D527E_C1581G	PIK3CA_E542VG_A1625TG
PIK3CA_E545D_G1635CT	PIK3CA_T1025SA_A3073TG
PIK3CA_Y1021C_A3062G	PIK3R1_N564K_C1693AG
PRKAG1_R70Q_G209A	AKT1_E17K_G49A
AKT1_K179M_A536T	BRAF_V600EAG_T1799ACG_R
CDK4_R24C_C70T	CDK4_R24H_G71A
CTNNB1_D32AGT_A95CGV	FBWX7_R465HL_G1394AT
FGFR3_G697C_G2089T	FGFR3_K650MT_A1949 TC
FGFR3_R248C_C742T	FGFR3_S371C_A1111T
FGFR3_Y373C_A1118G	GNAS_R201H_G602A
IDH1_R132HL_G395AT	KIT_N822YHD_A2464TCG
MET_R988C_C2962T	MET_Y1253D_T3757 G
NRAS_G13SRC_G37ACT	NRAS_Q61RPL_A182GCT
PIK3CA_Q546LPR_A1637TCG	TNK2_E346K_G1036A
PIK3CA_H1047RL_A3140 GT	ALK_F1245C_T3734G

The listed gene mutations were screened by Sequenom INT16/20 panel (Characterized Cell Line Core, MD Anderson Cancer Center) and *HRAS* was sequenced by Sanger sequencing. All examined loci were wild-type in the cSCC cell lines SRB1, SRB12, COLO16, and keratinocyte cell line HaCaT. The PIK3R1_M326I_G978 polymorphism was found in the SRB12 cell line.

Because the p38 and JNK stress-activated MAP kinases are well-established critical mediators of UV-induced apoptosis (Derijard et al., 1994; Chen et al., 1996; Tournier et al., 2000; Hildesheim et al., 2004), we explored the status of JNK and p38 activation by assessing phospho-JNK and phospho-p38 levels by Western blot (Figure 1F). Phospho-JNK levels in particular were highly upregulated upon UV irradiation and were significantly suppressed by treatment post-radiation with 1 μM PLX4720 in cSCC and HaCaT cell lines (Figure 1F). Similar effects were seen with 1 μM vemurafenib (data not shown) and in cells stressed with doxorubicin (Figure 1—figure supplement 2C). Importantly, ERK signaling remained intact, as evidenced both by the paradoxical activation of ERK (upregulation of phospho-ERK) and by the failure to upregulate BIM levels (Figure 1F,G). This pro-apoptotic BCL2 family member is upregulated by inhibition of ERK signaling (Collins et al., 2005) and in *BRAF*^*V600E*^ melanoma cells treated with vemurafenib (Paraiso et al., 2011). Since NOXA is a downstream effector of UV-induced apoptosis (Naik et al., 2007), we examined its expression and found that NOXA expression is induced by UV irradiation and suppressed by PLX4720 in all cell lines (Figure 1G), suggesting that inhibition of NOXA expression may be a mechanism of PLX4720-induced suppression of apoptosis. Finally, we examined the expression of the antiapoptotic BCL2 family member MCL1 because it is downregulated by UV exposure (Figure 1G), but as previously reported (Paraiso et al., 2011), unaffected by PLX4720 (Figure 1G).

To test the generality of these effects in cells in which ERK activity is suppressed by BRAFi, we extended our analysis to the BRAF^V600E^ melanoma cells A375 and WM35. As expected, phospho-ERK expression was strongly suppressed by PLX4720 (Figure 1H). Phospho-JNK and phospho-p38 were significantly upregulated following UV-irradiation (Figure 1H), showing that signaling to JNK and p38 is intact in *BRAF*^*V600E*^ melanoma cells. Here again, there was significant suppression of both phospho-p38 and phospho-JNK induction by PLX4720 (Figure 1H), and similar effects were seen with vemurafenib (data not shown).

**Figure 2.**
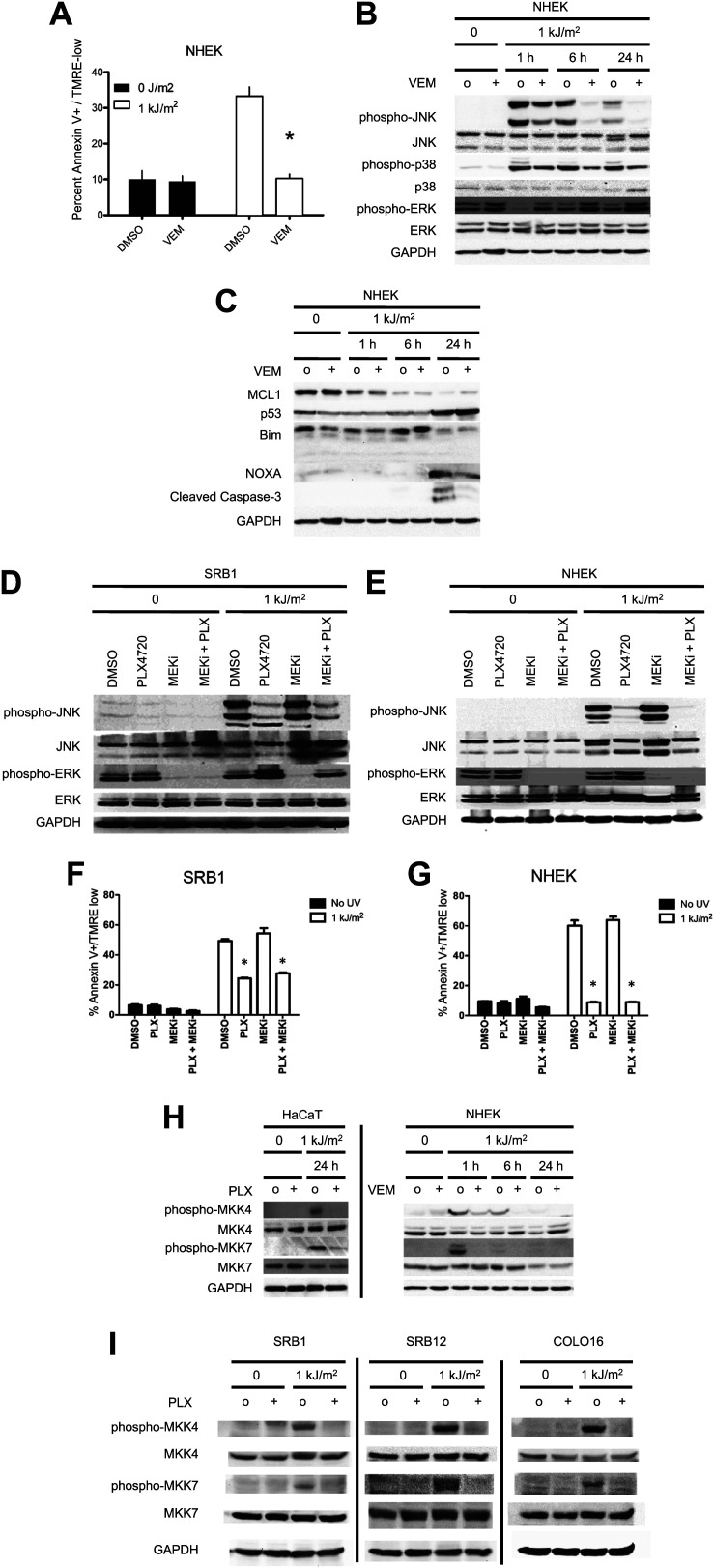
Vemurafenib and PLX4720 suppress apoptosis and JNK signaling in primary human keratinocytes and cSCC cells independently of MEK/ERK signaling. Normal human epidermal keratinocytes (NHEKs) were irradiated with 1 kJ/m^2^ of UVB in the absence (‘o’, 1:2000 DMSO) or presence (‘+’) of 1 μM vemurafenib and isolated for FACS analysis and protein extracts 24 hr later. (**A**) Apoptosis was significantly suppressed (70%) in the presence of vemurafenib as measured by FACS for Annexin V+, TMRE-low cells (n = 6, ‘*’ denotes statistical significance at p<0.05). (**B**) Western blot analysis showed induction of phospho-JNK and phospho-p38 within 1 hr following irradiation, which persisted for at least 24 hr and which was suppressed by vemurafenib at all time points. (**C**) MCL1 and BIM expression was not significantly modulated by vemurafenib; however, NOXA induction, which occurred at 24 hr, was reduced by vemurafenib. In these primary cells, p53 protein was stabilized by 24 hr and vemurafenib did not affect overall levels. Suppression of apoptosis, as measured by cleaved caspase-3 levels, was observed in the presence of vemurafenib-treated irradiated cells, consistent with the FACS results. To test the relevance of MEK signaling, cSCC (SRB1) and NHEK cells were irradiated with 1 kJ/m^2^ of UVB in the absence (‘o’, 1:2000 DMSO) or presence of 1 μM PLX4720 singly or in combination with 0.6 μM (NHEK) or 1.2 μM (SRB1) AZD6244 (MEKi) and isolated for FACS analysis and protein extracts 24 hr later. (**D**) SRB1 and (**E**) NHEK cells showed induction of phospho-JNK at 24 hr following irradiation, by Western in the presence (lane 7) and absence (lane 5) of MEKi. The addition of MEKi to PLX4720 did not affect the suppression of JNK activation (compare lanes 6, 8) despite potent suppression of phospho-ERK. (**F**) SRB1 and (**G**) NHEK cells exhibited a strong suppression of UV-induced apoptosis by PLX4720 (Annexin V+, TMRE-low cells; n = 6, ‘*’ denotes statistical significance at p<0.05) that was likewise unaffected by the addition of MEKi. To test whether upstream kinases in the JNK pathway were inhibited, MKK4 and MKK7 activation was probed in cells. (**H**) Both phospho-MKK4 and phospho-MKK7 were induced in HaCaT and NHEK cells following irradiation, and this was suppressed in the presence of 1 μM PLX4720 and vemurafenib, respectively. (**I**) In all cSCC cell lines, SRB1, SRB12, COLO16, phospho-MKK4 and phospho-MKK7 are strongly induced following irradiation, and this is suppressed in all lines by 1 μM PLX4720. **DOI:** http://dx.doi.org/10.7554/eLife.00969.008

We next examined the responses of primary normal human epidermal keratinocytes (NHEKs) to vemurafenib. UV-induced apoptosis was significantly suppressed (approximately 70%) by vemurafenib in these cells (Figure 2A, Figure 1—figure supplement 1D), and the UV-induced upregulation of phospho-JNK and phospho-p38 was likewise suppressed most significantly at 6 and 24 hr (Figure 2B). As in the cSCC and HaCaT cell lines, activation of ERK was observed following exposure to vemurafenib (Figure 2B). The presence of cleaved caspase-3 correlated with high levels of apoptosis in the UV-treated cells and its absence with rescue by vemurafenib at 24 hr post-irradiation (Figure 2C). In probing members of the BCL2 family, we found similar results to those in the cSCC and HaCaT cell lines. BIM and MCL1 were unaffected by vemurafenib but NOXA induction at 24 hr post-UV irradiation was diminished by vemurafenib (Figure 2C). The advantage of using primary cells is that *p53* is intact. In NHEKs, p53 is stabilized by 24 hr post-UV irradiation and this is unaffected by vemurafenib (Figure 2C). However, since BCL2 family members can be modulated by JNK (Tournier et al., 2000; Haeusgen et al., 2011) and p53 (Oda et al., 2000) in apoptosis, the inhibition of NOXA expression by PLX4720 and vemurafenib (Figures 1G and 2C) likely reflects *p53*-independent regulation of NOXA given that *p53* is mutant in HaCaT (Lehman et al., 1993) cells, p53 is undetectable in SRB12 cells, and p53 levels do not change with radiation in SRB1, COLO16, or HaCaT cells, (Figure 2—figure supplement 1). PUMA, BAX, BCL2, BCL-XL, and BCL2A1 expression were unchanged following irradiation and were unchanged by PLX4720 or vemurafenib exposure (data not shown, Figure 2—figure supplement 2). We conclude from our results that vemurafenib and PLX4720 suppress UV-induced apoptosis by inhibiting JNK signaling and NOXA induction in *BRAF* and *RAS* WT cells.

**Figure 2—figure supplement 1.**
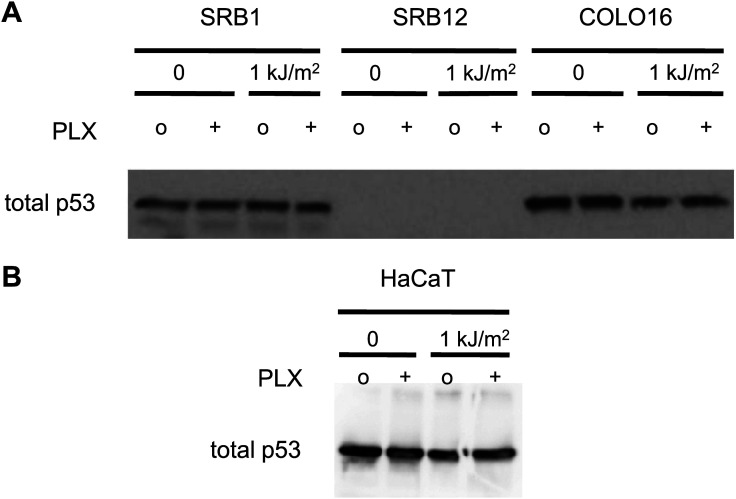
p53 does not respond to stress in cSCC and HaCaT cell lines. cSCC cell lines SRB1, SRB12, and COLO16 were either unirradiated or irradiated with 1 kJ/m^2^ of UVB in the absence (‘o’, 1:2000 DMSO) or presence (‘+’) of 1 μM PLX4720 and isolated for protein extracts 24 hr later. (**A**) Western blots of total p53 reveal that none of the cell lines upregulate p53 in response to UV irradiation. SRB12 cells do not express p53. (**B**) HaCaT cells are known to be mutant for *p53* and the presence of p53 in unstressed cells, combined with the failure to upregulate levels following UV radiation, is a hallmark of functionally inactive p53 in cell lines. Loading controls are the same as those in Figure 1F. **DOI:** http://dx.doi.org/10.7554/eLife.00969.009

**Figure 2—figure supplement 2.**
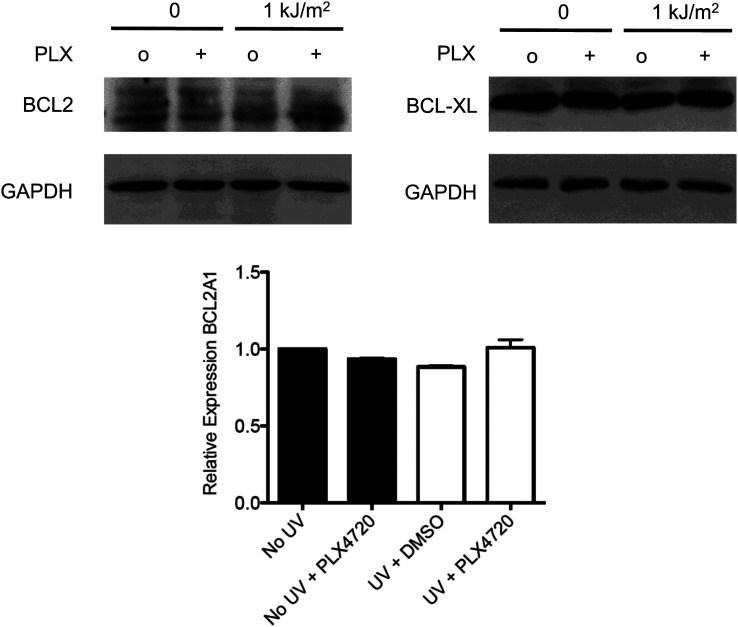
BCL2 family members BCL2, BCL-XL, and BCL2A1 are not modulated by acute UV exposure or PLX4720. HaCaT cells were either unirradiated or irradiated with 1 kJ/m^2^ of UVB in the absence (‘o’, 1:2000 DMSO) or presence (‘+’) of 1 μM PLX4720 and isolated for protein extracts 24 hr later. Western blots for BCL2 (2875P, Cell Signaling) and BCL-XL (2764P/clone 54H6, Cell Signaling) expression show that expression of neither is changed by acute UV exposure and or by PLX4720. c, qRT-PCR for BCL2A1 mRNA expression, referenced to GAPDH (Taqman) shows that BCL2A1 expression is likewise unchanged by acute UV exposure or by PLX4720. **DOI:** http://dx.doi.org/10.7554/eLife.00969.010

### BRAFi suppress JNK activity through off-target inhibition of ZAK, MKK4, MAP4K5

Although BRAFi-induced JNK inhibition is observed in BRAF-WT as well as BRAF^V600E^ cells (Figure 1F,H, 2B), with opposite effects on ERK signaling, we sought to further demonstrate that JNK inhibition and paradoxical ERK activation are independent and separable. We treated SRB1 and NHEK cells with the MEK inhibitor (MEKi) AZD6244 and PLX4720 singly and in combination with and without UV irradiation. While MEKi effectively abrogated ERK phosphorylation and activation (Figure 2D,E), this left PLX4720-mediated suppression of UV-induced JNK activation (Figure 2D,E) and apoptosis (Figure 2F,G) unaffected in both SRB1 and NHEK cells.

Because JNK and p38 isoforms are not significantly inhibited by PLX4720 or vemurafenib directly, (Tsai et al., 2008; Bollag et al., 2010) we probed the phosphorylation status of MKK4 and MKK7 (MAP2K7), the two proximal kinases that synergistically phosphorylate JNK and that are required for JNK activation (Tournier et al., 2001; Haeusgen et al., 2011). The phosphorylation of both MKK4 and MKK7, corresponding to their activation, was significantly upregulated in control UV-irradiated cells and inhibited by PLX4720 in all cSCC cell lines, HaCaT, and NHEK cells (Figure 2H,I).

**Figure 3.**
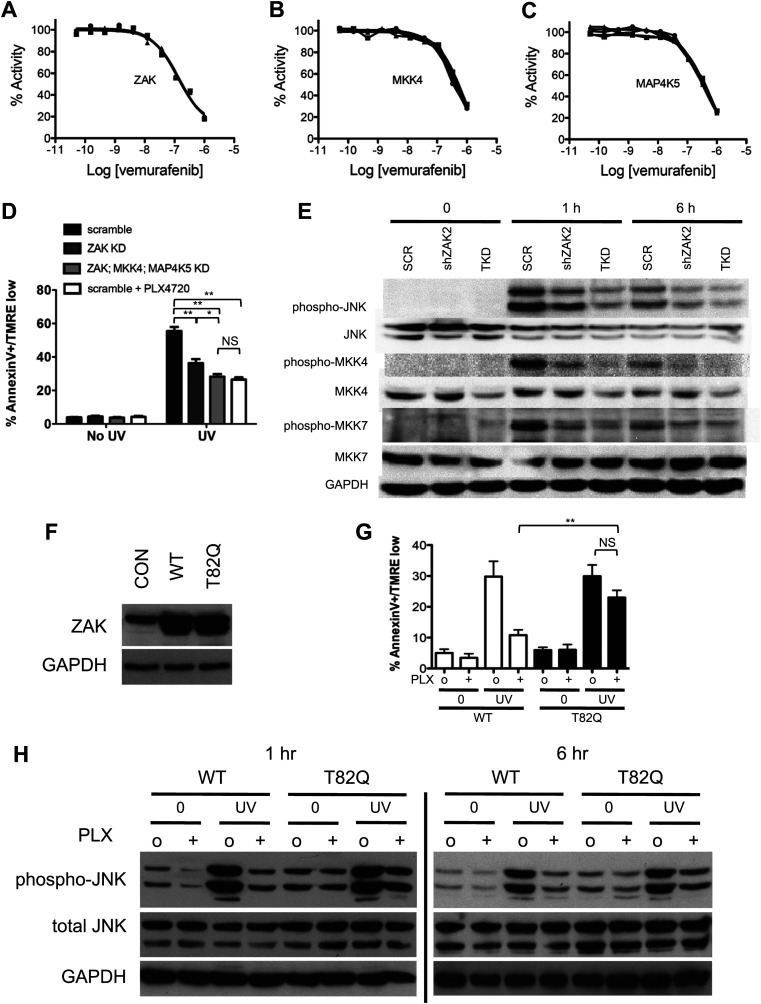
PLX4720 and vemurafenib suppress apoptosis and JNK signaling through inhibition of off-target kinases. (**A–C**) In-vitro kinase assays for ZAK, MKK4, and MAP4K5 were performed across a 10-point concentration range from 0.05 to 1000 nM in triplicate, revealing significant inhibition of kinase activity within the nM range for vemurafenib. (**D**) Lentiviral shRNA knockdown of ZAK singly or in combination with MKK4 and MAP4K5 (triple knockdown, ‘TKD’) was performed revealing potent suppression of apoptosis as measured by FACS for Annexin V+, TMRE-low cells (n = 5, ‘*’ denotes statistical significance at p<0.05, ‘**’ at p<0.01, ‘NS’ is not significant) at 24 hr following single dose UVB irradiation at 720 J/m^2^. ZAK knockdown and triple knockdown cells exhibit 70% and 94% suppression of apoptosis, respectively, relative to PLX4720-treated cells expressing a non-suppressing shRNA control (scramble, ‘SCR’). (**E**) Western blots of lysates obtained at 1 and 6 hr post-UV irradiation show potent induction of phospho-MKK4, phospho-MKK7, and phospho-JNK which are all suppressed with progressively increasing effect in ZAK single knockdown (‘shZAK2’) and triple knockdown (‘TKD’) HaCaT cells. (**F**) Western blots of HaCaT cells electroporated with pcDNA3-wild-type (WT) ZAK and the gatekeeper mutant pcDNA3-(T82Q) ZAK show equivalent expression. (**G**) HaCaT cells overexpressing ZAK (WT) and ZAK (T82Q) were irradiated with a single dose of UVB irradiation at 720 J/m^2^ in the absence (‘o’) and presence (‘+’) of 1 μM PLX4720 and apoptosis measured by FACS for Annexin V+, TMRE-low cells (n = 4, ‘**’ at p<0.01, ‘NS’ is not significant) at 24 hr. ZAK (WT) cells are sensitive to PLX4720-mediated suppression of apoptosis (bar 3 vs 4), but drug-treated ZAK (T82Q)-expressing cells undergo significantly more apoptosis than drug-treated ZAK (WT) cells (bar 4 vs 8), with bypass of PLX4720-induced suppression as compared to drug-treated ZAK (WT) cells (paired t-test, p=0.005). (**H**) Western blots of ZAK (WT) and ZAK (T82Q)-expressing HaCaT cells at 1 hr and 6 hr post-irradiation show that phospho-JNK activation is intact in both cell lines in the absence of drug (lanes 3, 7), but that drug-treated ZAK (T82Q)-expressing HaCaT cells have significantly more phospho-JNK activation at both 1 and 6 hr post-irradiation, as compared to drug-treated ZAK (WT)-expressing cells (lane 4 vs 8). **DOI:** http://dx.doi.org/10.7554/eLife.00969.013

We then performed a kinome screen of PLX4720 and vemurafenib against a panel of 38 kinases reported to be upstream of JNK (Keshet and Seger, 2010; Haeusgen et al., 2011) and other kinases previously tested against PLX4720 and vemurafenib (Tsai et al., 2008; Bollag et al., 2010) using a quantitative competitive binding assay (Davis et al., 2011) at four concentrations (50 nM, 200 nM, 1 μM, 10 μM). We extended previously reported results obtained on this platform (Davis et al., 2011) by testing a wider concentration range and by additionally testing vemurafenib. Reported biochemical IC50s for vemurafenib (Bollag et al., 2010) and PLX4720 (Tsai et al., 2008) against multiple kinases including BRAF^V600E^, MAP4K5, SRMS, and BRK were quantitatively similar to the estimated *K*_*d*_, confirming the validity of this assay (Tables 2 and 3). We confirmed that ZAK and MKK4 (MAP2K4) have high binding affinities comparable to that of the intended target, BRAF (estimated *K*_*d*_ below 50 nM) for both PLX4720 (Davis et al., 2011) and vemurafenib, and confirmed activity against MAP4K5 (Bollag et al., 2010) (Tables 2 and 3). To demonstrate an effect on activity, in vitro kinase assays were performed (Figure 3A–C) and revealed biochemical IC50s of 187 ± 5 nM, 460 ± 41 nM, and 354 ± 26 nM for ZAK, MKK4, and MAP4K5, respectively. All of these values are within the range of reported correspondences between binding assays and activity-based assays and with reported data (Anastassiadis et al., 2011; Davis et al., 2011). Importantly, at 1 μM vemurafenib used in our experiments, the residual activity of ZAK, MKK4, and MAP4K5 kinases, was 18.9 ± 0.5%, 29.6 ± 1.1%, and 25.7 ± 0.6%, respectively.

**Table 2.** Quantitative competitive binding assays reveal additional kinase targets of PLX4720 **DOI:** http://dx.doi.org/10.7554/eLife.00969.011

Gene Name	Entrez gene Symbol	Percent control (50 nM)	Percent control (200 nM)	Percent control (1000 nM)	Percent control (10 μM)	Calculated estimate of IC50 (nM)	Published biochemical IC50 (nM)
ASK1	MAP3K5	89	98	97	100	14,179.29	
ASK2	MAP3K6	94	100	100	100		
BLK	BLK	91	78	32	1	446.56	
**BRAF(V600E)**	**BRAF**	**38**	**19**	**3.9**	**0.1**	**32.04**	**13**
BRK	PTK6	47	14	2.4	0.2	30.38	130
DLK	MAP3K12	95	98	100	92		
FGR	FGR	69	38	11	2.5	153.47	
HPK1	MAP4K1	100	100	100	47		
LZK	MAP3K13	94	100	96	75		
MAP3K1	MAP3K1	96	100	92	84		
MAP3K15	MAP3K15	94	97	91	59		
MAP3K2	MAP3K2	100	93	87	41		
MAP3K3	MAP3K3	94	97	98	75		
MAP3K4	MAP3K4	100	100	100	65		
MAP4K2	MAP4K2	98	100	99	67		
MAP4K3	MAP4K3	100	95	90	56		
MAP4K4	MAP4K4	92	99	100	46		
**MAP4K5**	**MAP4K5**	**96**	**100**	**63**	**8**	**1257.42**	
MEK3	MAP2K3	100	100	100	64		
**MEK4**	**MAP2K4**	**48**	**27**	**2.6**	**0.05**	**37.96**	
MEK6	MAP2K6	82	100	100	47		
MINK	MINK1	89	100	98	55		
MKK7	MAP2K7	100	100	100	84		
MLK1	MAP3K9	100	100	100	100	>10,000	>5000
MLK2	MAP3K10	100	82	100	76		
MLK3	MAP3K11	100	100	100	100		
MST1	STK4	100	93	84	55	6709.79	>5000
OSR1	OXSR1	100	94	95	42		
PAK1	PAK1	93	97	83	22		
RIPK1	RIPK1	99	87	85	50		
SRMS	SRMS	1.9	0.55	0.05	0	0.64	
STK39	STK39	100	100	100	59		
TAK1	MAP3K7	90	88	85	49		
TAOK1	TAOK1	87	94	89	65	7532.57	>5000
TAOK2	TAOK2	92	100	93	51		
TAOK3	TAOK3	100	98	96	58		
TNIK	TNIK	97	89	79	24		
**ZAK**	**ZAK**	**20**	**4**	**0.7**	**0.1**	**9.47**	

Quantitative competitive binding assays were performed for a group of kinases previously tested against PLX4720 as well as a group of MAP kinases upstream of JNK. Published biochemical IC50s for PLX4720 are listed (see main text) for comparison and demonstrate good quantitative correspondence between estimated *K*_*d*_ from binding assays and biochemical IC50s. ZAK and MKK4 (MAP2K4) were very tightly bound by PLX4720 with estimated *K*_*d*_ below 50 nM. Bold text indicates the kinases tested for inhibition by PLX4720 with in-vitro kinase assays.

**Table 3.** Quantitative competitive binding assays reveal additional kinase targets of vemurafenib **DOI:** http://dx.doi.org/10.7554/eLife.00969.012

		Percent control (50 nM)	Percent control (200 nM)	Percent control (1000 nM)	Percent control (10 μM)	Calculated estimate of IC50 (nM)	Published biochemical IC50 (nM)
ASK1	MAP3K5	90	94	97	100	11,972.22	>1000
ASK2	MAP3K6	94	98	100	74		
BLK	BLK	96	66	30	0.55	518.03	547
**BRAF(V600E)**	**BRAF**	**63**	**25**	**5.4**	**0.5**	**64.78**	**31**
BRK	PTK6	63	28	6.9	0.35	68.04	213
DLK	MAP3K12	98	97	66	92		
FGR	FGR	65	49	13	1.6	149.26	63
HPK1	MAP4K1	95	88	67	15		
LZK	MAP3K13	100	99	93	74		
MAP3K1	MAP3K1	98	84	89	81		
MAP3K15	MAP3K15	84	100	84	91		
MAP3K2	MAP3K2	91	91	89	83		
MAP3K3	MAP3K3	87	97	100	94		
MAP3K4	MAP3K4	95	92	87	46		
MAP4K2	MAP4K2	99	82	95	46		
MAP4K3	MAP4K3	80	90	82	24		
MAP4K4	MAP4K4	96	92	83	23	2842.34	>1000
**MAP4K5**	**MAP4K5**	**62**	**33**	**4.1**	**0.1**	**58.21**	**51**
MEK3	MAP2K3	100	96	98	54		
**MEK4**	**MAP2K4**	**19**	**4.1**	**0.2**	**0.05**	**6.82**	
MEK6	MAP2K6	91	97	87	21	4080.69	>1000
MINK	MINK1	100	100	91	66	14,761.44	>1000
MKK7	MAP2K7	97	95	94	85		
MLK1	MAP3K9	100	93	97	41	13,979.88	>1000
MLK2	MAP3K10	92	96	87	78		
MLK3	MAP3K11	98	100	100	77		
MST1	STK4	99	83	51	12		
OSR1	OXSR1	100	100	89	98		
PAK1	PAK1	99	98	91	46		
RIPK1	RIPK1	92	100	99	73		
SRMS	SRMS	24	9.6	0.75	0	11.15	18
STK39	STK39	100	100	97	66		
TAK1	MAP3K7	93	88	86	88		
TAOK1	TAOK1	91	100	97	79		
TAOK2	TAOK2	98	92	95	70	11,770.83	>1000
TAOK3	TAOK3	92	98	92	80	15,468.75	>1000
TNIK	TNIK	95	94	66	11		
**ZAK**	**ZAK**	**9**	**1.8**	**0.25**	**0.05**	**4.03**	

Quantitative competitive binding assays were performed for a group of kinases previously tested against vemurafenib as well as a group of MAP kinases upstream of JNK. Published biochemical IC50s for vemurafenib are listed (see main text) for comparison and demonstrate good quantitative correspondence between estimated *K*_*d*_ from binding assays and biochemical IC50s. ZAK and MKK4 (MAP2K4) were very tightly bound by vemurafenib with estimated *K*_*d*_ below 50 nM. Bold text indicates the kinases tested for inhibition by vemurafenib with in-vitro kinase assays.

**Figure 3—figure supplement 1.**
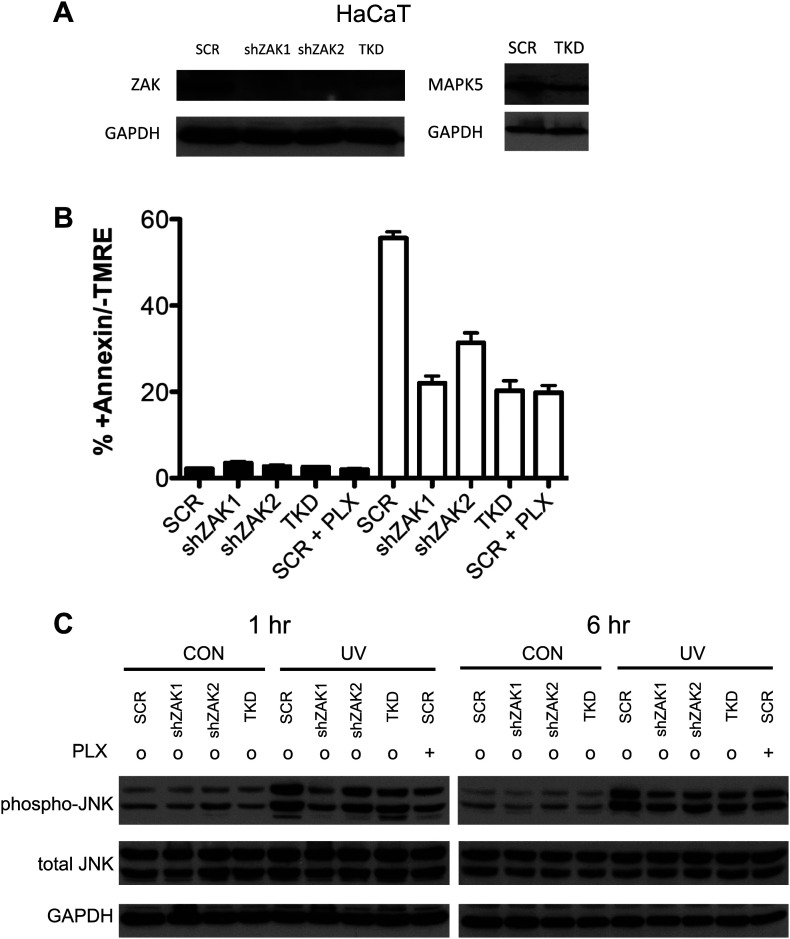
Knockdown of ZAK potently inhibits JNK activation and UV-induced apoptosis. (**A**) Western blot of HaCaT cells expressing two shRNA clones and HaCaT TKD cells (containing shZAK2) all show significant knockdown of ZAK protein, though shZAK2 produces slightly less knockdown. Approximately 54.6% knockdown (ImageJ) of MAP4K5 is observed in TKD cells. (**B**) HaCaT cells, expressing non-silencing scramble-shRNA (‘SCR’), shZAK1, shZAK2, or TKD, were either unirradiated (black bars) or irradiated (open bars) with 1 kJ/m^2^ of UVB in the absence (‘o’, 1:2000 DMSO) or presence (‘+’) of 1 μM PLX4720 and analyzed by FACS for apoptosis (Annexin V+, TMRE-) at 24 hr. UV-induced apoptosis is significantly suppressed by both ZAK shRNA clones in HaCaT cells and in TKD cells. The shZAK2 clone, which results in less knockdown than shZAK1, produces correspondingly less suppression of UV-induced apoptosis (93.7% for shZAK1 vs 67.8% for shZAK2). shZAK1-expressing HaCaT cells, TKD cells, and PLX4720-treated HaCaT scrambled-shRNA-expressing cells show similar degrees of suppression, again consistent with the fact that ZAK can account for the majority of the effect of BRAFi-induced suppression of JNK signaling. (**C**) HaCaT cells, treated as above, were processed for Western blots at 1 and 6 hr following UV exposure to assess JNK activation. Significant suppression of phospho-JNK is observed at 1 hr and 6 hr post-irradiation in all cell lines where ZAK is knocked down, as well as TKD cells and SCR cells treated with PLX4720. In comparing the shZAK1 and shZAK2-expressing HaCaT cells, the degree of phospho-JNK inhibition correlates exactly with the degree of knockdown of ZAK particularly at 1 hr: less phospho-JNK inhibition is observed with less ZAK knockdown. **DOI:** http://dx.doi.org/10.7554/eLife.00969.014

**Figure 3—figure supplement 2.**
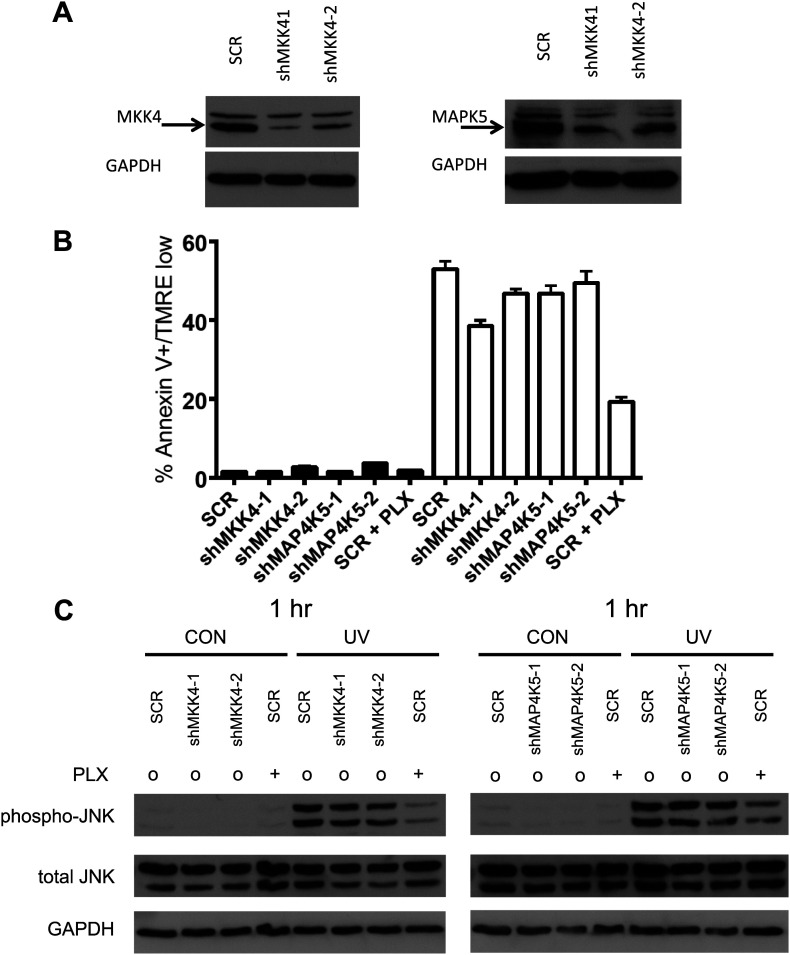
Single knockdown of MKK4 or MAP4K5 partially inhibits JNK activation and UV-induced apoptosis. (**A**) Western blot of HaCaT cells expressing two shRNA clones against MKK4 and MAP4K5 show significant knockdown of targets proteins (shMKK4-1: 89.3%, shMKK4-2: 71.9%, shMAP4K5-1: 86.4%, shMAP4K5-2: 84.1%; ImageJ). (**B**) HaCaT cells, expressing non-silencing scramble-shRNA (‘SCR’), shMKK4-1, shMKK4-2, shMAP4K5-1, or shMAP4K5-2 were either unirradiated (black bars) or irradiated (open bars) with 720 J/m^2^ of UVB in the absence (‘o’, 1:2000 DMSO) or presence (‘+’) of 1 μM PLX4720 and analyzed by FACS for apoptosis (Annexin V+, TMRE-) at 24 hr. UV-induced apoptosis is suppressed most substantially by MKK4 (up to 27.3%), but not substantially by MAP4K5 (up to 11.6%) in HaCaT cells. These results are consistent with the fact that ZAK can account for the majority of the effect of BRAFi-induced suppression of JNK signaling. Importantly, since MKK4 is important for JNK activation, and ZAK activates MKK4, the partial suppression of phospho-JNK activation and apoptosis is expected. (**C**) HaCaT cells, treated as above, were processed for Western blots at 1 hr following UV exposure to assess JNK activation. Significant activation of phospho-JNK is still observed at 1 hr post-irradiation in all cell lines, as compared to SCR cells treated with PLX4720. **DOI:** http://dx.doi.org/10.7554/eLife.00969.015

**Figure 3—figure supplement 3.**
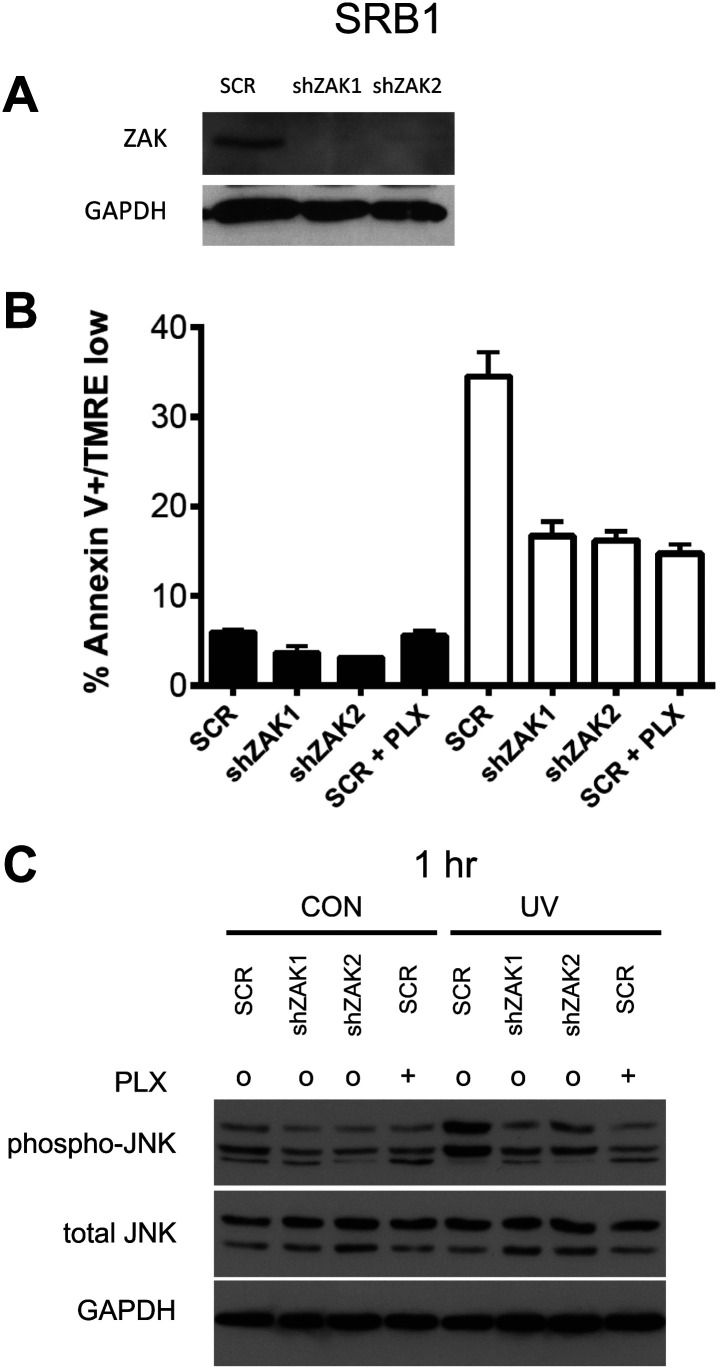
Knockdown of ZAK potently inhibits JNK activation and UV-induced apoptosis in SRB1 cells. (**A**) Western blot of SRB1 cells expressing two shRNA clones (shZAK1, shZAK2) all show significant knockdown of ZAK protein. (**B**) SRB1 cells, expressing non-silencing scramble-shRNA (‘SCR’), shZAK1, or shZAK2, were either unirradiated (black bars) or irradiated (open bars) with 720 J/m^2^ of UVB in the absence (‘o’, 1:2000 DMSO) or presence (‘+’) of 1 μM PLX4720 and analyzed by FACS for apoptosis (Annexin V+, TMRE-) at 24 hr. UV-induced apoptosis is significantly suppressed by both ZAK shRNA clones in SRB1 cells. shZAK1/2-expressing SRB1 cells and PLX4720-treated SRB1 scrambled-shRNA-expressing cells show similar degrees of suppression (90%, 92.5% of drug-treated cells), again consistent with the fact that ZAK can account for the majority of the effect of BRAFi-induced suppression of JNK-dependent apoptosis. (**C**) SRB1 cells, treated as above, were processed for Western blots at 1 hr following UV exposure to assess JNK activation. Significant suppression of phospho-JNK is observed at 1 hr post-irradiation in all cell lines where ZAK is knocked down, as well as in SCR cells treated with PLX4720. **DOI:** http://dx.doi.org/10.7554/eLife.00969.016

**Figure 3—figure supplement 4.**
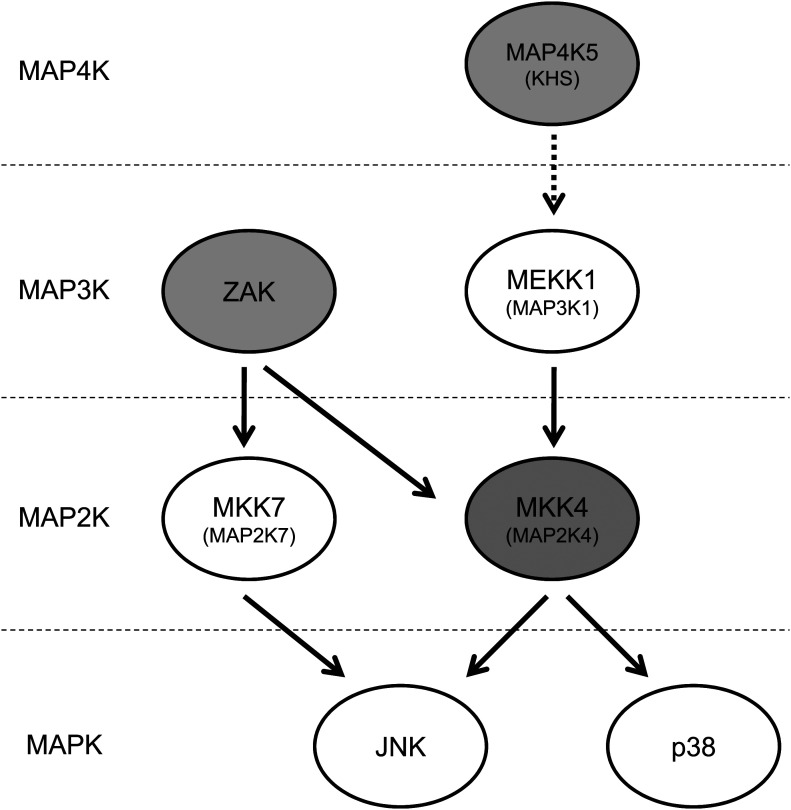
Vemurafenib and PLX4720 inhibit multiple kinases upstream of JNK and p38. The schematic shows MAP kinases upstream of JNK and p38 that are inhibited by these BRAF inhibitors (gray-shaded). Vemurafenib and PLX4720 inhibit ZAK (principally) and MKK4 (MEK4/MAP2K4), resulting in inhibition of MKK7 and MKK4 and, ultimately, JNK. p38 activation was diminished by drug exposure in some contexts , but not to the degree that JNK activation was. Vemurafenib and PLX4720 also inhibit MAP4K5, which has been shown to be upstream of MKK4 and JNK. **DOI:** http://dx.doi.org/10.7554/eLife.00969.017

To examine the requirements for ZAK, MAP4K5, and MKK4 in activating JNK activation and apoptosis more directly, we performed lentiviral shRNA knockdown experiments in HaCaT cells. HaCaT cells with knockdown of ZAK (‘shZAK2’) showed a strong suppression of ZAK protein expression (Figure 3—figure supplement 1A) and of UV-induced apoptosis, showing 70% suppression of apoptosis relative to that achieved by PLX4720 in control (‘SCR’) cells (Figure 3D, Figure 3—figure supplement 1A). An additional clone of shRNA against ZAK (‘shZAK1’) showed similar results (Figure 3—figure supplement 1B,C), demonstrating that even greater knockdown of ZAK can account for nearly the entire effect of PLX4720 on JNK activation and apoptosis. Western blots show significant suppression of phospho-MKK4/MKK7 in shZAK2 knockdown cells (Figure 3E). Triple knockdown cells (‘TKD’) with combined shRNA knockdown of ZAK, MKK4, and MAP4K5 kinases, as confirmed by Western (Figure 3E, Figure 3—figure supplement 1A), showed comparable suppression of apoptosis to that of drug-treated control cells (Figure 3D, Figure 3—figure supplement 1B) and substantial suppression of phospho-MKK4/MKK7 induction (Figure 3E). Furthermore, single knockdown of MKK4 and MAP4K5 (Figure 3—figure supplement 2A), only partially suppresses UV-induced apoptosis or phospho-JNK induction in HaCaT cells (Figure 3—figure supplement 2B,C). Knockdown of ZAK alone was able to account for 91.3% of the suppression of UV-induced apoptosis in a distinct cell line, SRB1 (Figure 3—figure supplement 3A,B), with corresponding suppression of phospho-JNK induction (Figure 3—figure supplement 3C). As knockdown of ZAK alone can account for up to 93.7% of the effect of PLX4720 treatment, we conclude that the potent inhibition of JNK activation and resultant apoptosis by PLX4720 and vemurafenib is due to the off-target inhibition of ZAK principally, with smaller additional contributions from inhibition of MKK4 and MAP4K5, which abrogates the activation of the two kinases essential for JNK phosphorylation and activation: MKK4 and MKK7 (Figures 2H–I,3D–E, Figure 3—figure supplement 4). Consistent with our findings, ZAK has been shown to be critically important for JNK activation upstream of MKK4 and MKK7 (Wang et al., 2005) and doxorubicin-induced apoptosis (Sauter et al., 2010; Wong et al., 2013).

### Expression of gatekeeper mutant ZAK reverses BRAFi-mediated apoptosis suppression

Our biochemical and shRNA data showed that knockdown of ZAK suppressed phospho-JNK activation and apoptosis (Figure 3D–E, Figure 3—figure supplements 1,3) and that the degree of knockdown correlated with the degree of JNK and apoptosis suppression. To show that PLX4720 suppresses apoptosis primarily through direct action on ZAK in cells, we employed a chemical-genetic approach by engineering a gatekeeper mutant ZAK (T82Q). Gatekeeper mutant kinases, in which the threonine (T) is replaced by a larger amino acid, in our case glutamine (Q), are often rendered insensitive to small molecule inhibitors and are an important mechanism of drug resistance (Daub et al., 2004; Whittaker et al., 2010). We overexpressed equivalent amounts of ZAK (T82Q) wild-type ZAK (WT) in HaCaT cells (Figure 3F), and compared their UV responses.

Whereas ZAK (WT) cells were sensitive to PLX4720-mediated suppression of apoptosis (Figures 1D and 3G), drug-treated ZAK (T82Q)-expressing cells underwent 2.13-fold more apoptosis than drug-treated ZAK (WT) cells (bar 4 vs 8; p=0.005), corresponding to 76.9% of the levels of apoptosis in untreated cells (bars 3, 7 vs 8; p=0.08) (Figure 3G). The effects on apoptosis corresponded to higher levels of phospho-JNK, even in drug-treated cells expressing the ZAK (T82Q) mutant as compared to drug-treated ZAK (WT)-expressing cells at both 1 hr and 6 hr post-irradiation (lane 4 vs 8; Figure 3H). Sustained activation of JNK is necessary for apoptosis (Tobiume et al., 2001; Kamata et al., 2005; Ventura et al., 2006), and our results show that PLX4720-treated ZAK (T82Q)-expressing cells retain higher activation across 1–6 hr as compared to PLX4720-treated ZAK (WT) cells.

### Vemurafenib suppresses JNK activity and apoptosis in cSCC arising in treated patients

**Figure 4.**
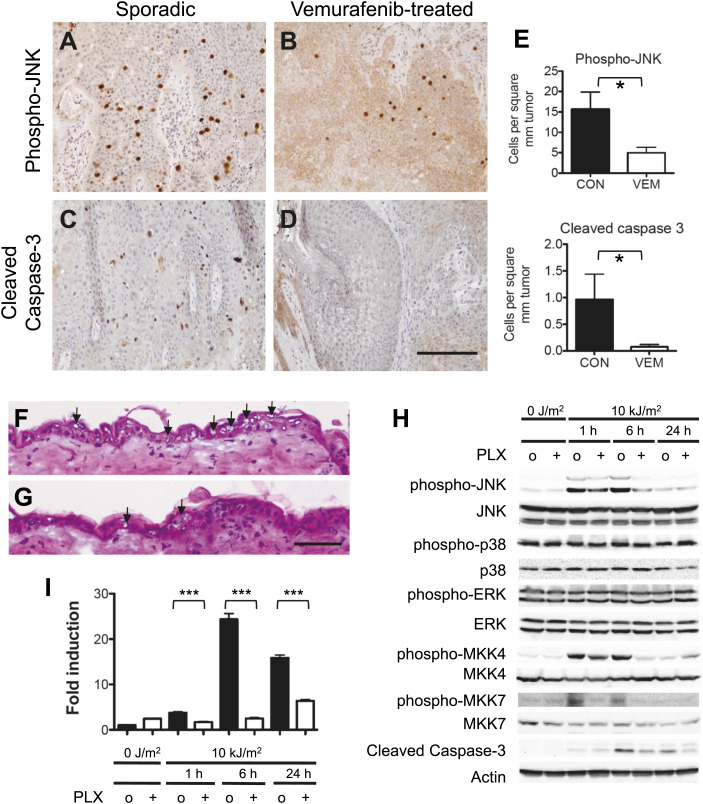
Vemurafenib and PLX4720 suppress apoptosis and JNK signaling in vivo. (**A–D**) cSCC samples from vemurafenib-treated patients and non-treated patients were analyzed by immunohistochemistry for phospho-JNK and cleaved caspase-3 expression. cSCC arising in vemurafenib-treated patients show decreased expression of phospho-JNK (**B**) and cleaved caspase-3 (**D**) as compared to sporadic cSCC in patient never treated with vemurafenib (**A** and **C**). Scale bar is 100 μm. (**E**) Comparisons of stained cells normalized to mm^2^ of tumor area revealed significant suppression of both phospho-JNK and cleaved caspase 3 expression in vemurafenib-treated cSCC (‘*’, p<0.05). (**F** and **G**) Hematoxylin-stained cryosections of skin harvested at 24 hr post-irradiation showed extensive apoptosis (arrowheads) with vacuolated blebbed cells and clumped pyknotic nuclei in control-treated mice (**F**) and significantly fewer apoptotic cells in PLX4720-treated mice (**G**). Scale bar is 50 μm. (**H–I**) Vehicle-treated (‘o’) and PLX4720-treated (‘+’) mice were unirradiated or irradiated once, and epidermis was harvested at 1 hr, 6 hr, and 24 hr post-irradiation. (**H**) Significant UV-induced upregulation of both phospho-JNK and phospho-p38 were observed within 1 hr, with significant suppression of phospho-JNK in PLX4720-treated mice by 6 hr and minimal suppression of phospho-p38. Phospho-ERK levels remained constant. The upstream regulators of JNK, MKK4 and MKK7, were both significantly activated within 1 hr of irradiation, and potently suppressed in PLX4720-treated mice. Cleaved caspase-3 levels increased within 6 hr and were suppressed in PLX4720-treated mice. (**I**) Noxa was induced most significantly at 6 hr and was potently suppressed by PLX4720 at all time points (‘***’, p<0.001). **DOI:** http://dx.doi.org/10.7554/eLife.00969.018

We then explored whether vemurafenib or PLX4720-mediated suppression of JNK and apoptosis is relevant in vivo. We first examined cSCC arising in patients treated with vemurafenib and compared them to sporadic cSCC that were histologically similar, arising in individuals never treated with vemurafenib (Figure 4A–E). Phospho-JNK and cleaved caspase-3 expression were assessed by immunohistochemistry and then quantified following normalization by unit area (mm^2^) of tumor tissue (malignant keratinocytes) only (Figure 4A–D, Figure 4—figure supplement 1). Sporadic cSCC arising in patients never treated with vemurafenib (n = 15) contained substantially greater expression of phospho-JNK (p=0.013; Figure 4A,E) and cleaved caspase-3 (p=0.042; Figure 4C,E) as compared to lesions arising in vemurafenib-treated patients (n = 16; Figure 4B,D,E). Therefore, we found significant reductions in phospho-JNK and cleaved caspase-3 expression in human cSCC suggesting that suppression of JNK activity and apoptosis occur in vivo in patients treated with vemurafenib.

**Figure 4—figure supplement 1.**
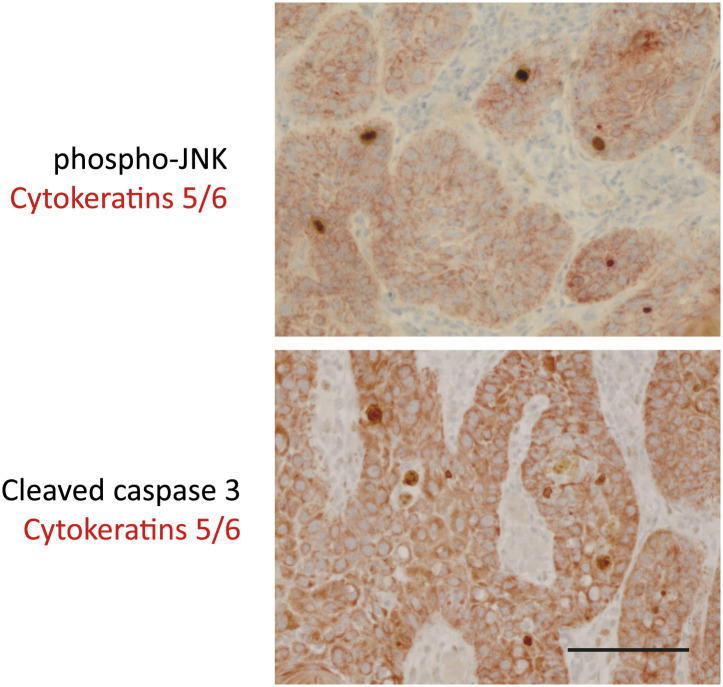
Double staining of sporadic cSCC confirms phospho-JNK and cleaved caspase-3 expression within keratinocytes of tumors. Sections were processed for standard immunohistochemistry and stained with primary antibodies against phospho-JNK, cleaved caspase-3 (Cell Signaling; peroxidase–DAB) as before, together with antibodies against cytokeratins 5/6 (clone D5/16 B4—Thermo; peroxidase–AEC). Results of the double staining show that in all cases, phospho-JNK staining and cleaved caspase 3 staining was observed exclusively in keratinocytes within tumors. Keratinocytes (CK5/6 +) are significantly larger and have more cytoplasm than macrophages or lymphocytes. Scale bar is 50 μM. **DOI:** http://dx.doi.org/10.7554/eLife.00969.019

### BRAFi suppress acute UVR-induced epidermal apoptosis in vivo

We then probed the acute, in vivo, short-term UV response in skin by pre-treating C57BL/6 mice with PLX4720 administered by oral gavage 40–80 mg/kg twice a day for 2–4 days (Tsai et al., 2008). Following depilation, mice were irradiated once using a solar simulator (Oriel) with 10 kJ/m^2^ UVB. The skin was harvested at 1 hr, 6 hr, and 24 hr post-irradiation. Consistent with our other results, we found significant apoptosis of epidermal keratinocytes in irradiated mouse skin that was suppressed by PLX4720 treatment (12.7 ± 0.4 apoptotic cells/mm vs 4.9 ± 0.3 apoptotic cells/mm skin, [n = 3 pairs], p<10^−5^; Figure 4F,G), a finding corroborated by cleaved caspase-3 levels, which were induced within 6 hr of irradiation and suppressed in PLX4720-treated mice (Figure 4H). As expected, phospho-JNK and phospho-p38 were significantly upregulated following UV irradiation and phospho-JNK was significantly suppressed by PLX4720 (Figure 4H). The upstream kinases MKK4 and MKK7 were likewise activated by UV radiation and suppressed by PLX4720 at 1 and 6 hr post-irradiation, confirming the importance of this mechanism of PLX4720-induced JNK signaling suppression in vivo. Finally, Noxa mRNA expression, as measured by qPCR, was strongly induced by UV exposure, a response significantly dampened by PLX4720 treatment (Figure 4I).

### BRAFi accelerates UVR-driven cSCC development in Hairless mice

**Figure 5.**
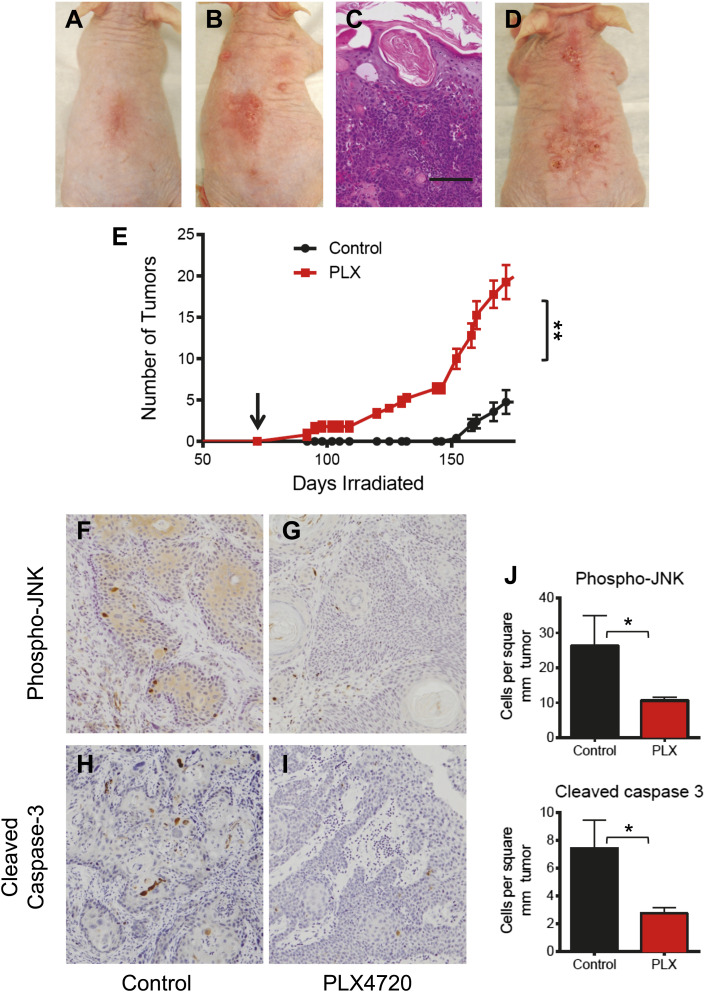
PLX4720 and JNK inhibition dramatically accelerate cSCC development in the UV-driven Hairless mouse model. (**A–E)** Chronically-irradiated Hairless mice were treated with PLX4720 (n = 5), or vehicle (n = 5) starting at day 72 (arrow, **E**). Tumors were induced within 20 days of PLX-4720 treatment (**B**), whereas only erythema was seen in control animals (**A**). The tumors in PLX4720-treated mice progressed to well-differentiated cSCC (**C**, scale bar 75 μm), steadily increasing in size and number (**D**, day 132). **(E)** Even at 150 days (78 days of drug treatment), only PLX4720-treated mice had tumors and the differences in tumor number persisted throughout (‘**’, p=0.0026). **(F–J)** cSCC from mice were harvested and assessed for phospho-JNK and cleaved caspase 3 expression by immunohistochemistry. Tumors from PLX4720-treated animals showed significantly lower levels of phospho-JNK (**G**) and cleaved caspase 3 (**I**) as compared to control-treated animals (**F** and **H**). Differences in these parameters were significant across all comparisons (**J**, ‘*’, p<0.05). **DOI:** http://dx.doi.org/10.7554/eLife.00969.020

We also used the Hairless mouse model of squamous cell carcinoma to assess whether PLX4720 would affect UV-driven tumor development. This is particularly relevant since it appears that UV exposure is an important initiating event in BRAFi-accelerated cSCC (Su et al., 2012). Unlike the DMBA/TPA model, in which lesions almost universally harbor *Hras* mutations (Brown et al., 1990), the Hairless model has a very low frequency of *Ras* mutation in papillomas and carcinomas (van Kranen et al., 1995), more similar to sporadic human cSCC. The cohorts (n = 5 each) were identically irradiated thrice weekly (12.5 kJ/m^2^ per week UVB) for 72 days before starting on PLX4720 treatment vs vehicle control. Within 20 days of administration of drug, hyperkeratotic papules were visible on the backs of PLX4720-treated animals (Figure 5A,B), which steadily grew into cSCC over the following several weeks (Figure 5C,D). Within this period of 150 days (78 days of drug treatment), control-treated mice had not yet developed any visible lesions (Figure 5E). When we quantified the effects of each of these drug treatments, we found significant decreases in both phospho-JNK expression (p=0.046; Figure 5F,G,J) and cleaved caspase 3 expression (p=0.019; Figure 5H–J) in PLX4720-treated mice as compared to control-treated mice. Importantly, we sequenced the entire coding regions for *Ras* (*Hras*, *Kras*, *Nras*) and found no mutations in any of the tumors in PLX4720-treated mice, as compared to one of 14 papillomas and carcinomas in a cohort of control-treated chronically-irradiated Hairless mice (Figure 5—figure supplement 1).

**Figure 5—figure supplement 1.**
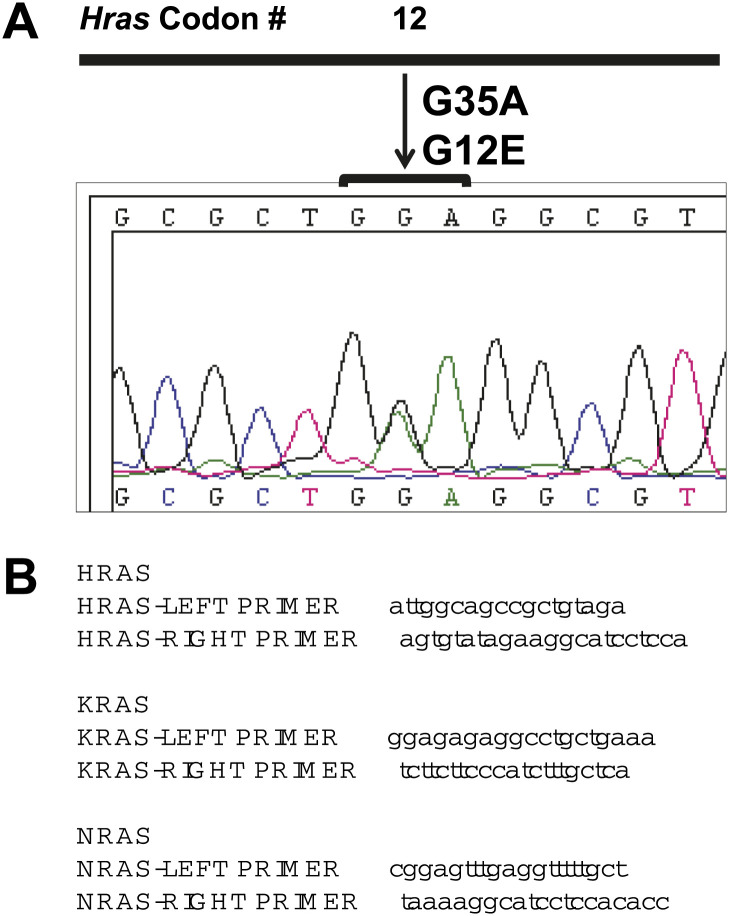
cSCC and papillomas arising in Hairless mice treated with PLX4720 do not have *Ras* mutations. (**A** and **B**) cDNA was reverse-transcribed from total RNA, PCR-amplified with the above primers (**B**) and analyzed by Sanger sequencing for mutations in both directions. No mutations in *Hras*, *Kras*, or *Nras* were detected in any of the papillomas (n = 5) or carcinomas (n = 3) isolated from PLX4720-treated mice. One of the papillomas from untreated mice had a heterozygous point mutation (**A**) in *Hras* (G35A, G12E) among 14 samples (12 papillomas, 2 cSCC). **DOI:** http://dx.doi.org/10.7554/eLife.00969.021

### Paradoxical ERK activation and off-target JNK inhibition cooperate to accelerate tumor growth

**Figure 6.**
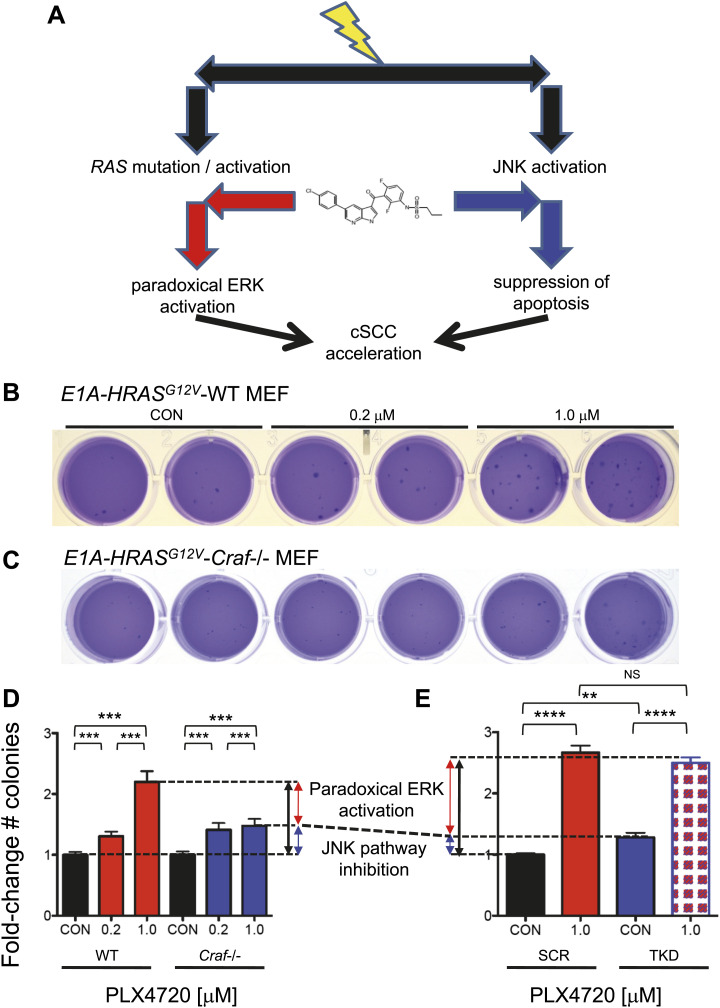
Paradoxical ERK activation and JNK pathway inhibition make significant and separable contributions to BRAFi-induced growth. (**A**) We envision two separable, parallel mechanisms by which PLX4720 and vemurafenib contribute to cSCC development. Drug-induced paradoxical ERK activation and inhibition of JNK signaling occur in parallel, but the former depends on intact *CRAF*. (**B** and **C**) Representative soft agar colonies of E1A and *HRAS*^*G12V*^-transformed wild-type (WT) (**B**) and *Craf*−/− (**C**) MEFs, following exposure to 0.2 μM and 1.0 μM PLX4720 over 4–6 weeks show significant colony-forming advantages conferred by BRAFi. (**D**) The fold-change in colony counts of transformed wild-type (WT) (n = 22 replicates) and *Craf*−/− (n = 14 replicates) MEFs demonstrate a dose-dependent increase in colonies, particularly for WT MEFs. The difference between colony formation advantages conferred by 1.0 μM PLX4720 in WT vs *Craf*−/− MEFs was interpreted to reflect the contribution of paradoxical ERK signaling (red arrow), which depends upon *Craf*, and is 60% of the total effect (black arrow), with the remainder composed of other effects including JNK inhibition (blue arrow). All differences between each MEF population were significant (‘***’, p<0.001) (**E**) The fold-change in colony counts of transformed HaCaT cells with (‘TKD’) and without (‘SCR’) triple lentiviral shRNA knockdown of ZAK, MAP4K5, and MAP2K4, show significant differences between 1.0 μM PLX4720-treated and control-treated conditions (‘****’, p<10^−10^). Importantly, untreated TKD cells had a significant advantage over untreated SCR HaCaT cells (‘**’, p<0.01), which we interpreted to be the contribution of JNK signaling inhibition, of 17.6% (blue arrow). Drug-treated SCR and TKD cells both had a similar degree of total colony formation advantage (averaged as black arrow), as expected, since the TKD cells are not expected to have any additional suppression of JNK signaling in the presence of drug (‘NS’, p=0.17, Figure 3D). Therefore, the colony counts for these two distinct systems (**D** and **E**), when taken together, show that JNK pathway inhibition accounts for approximately 17.6–40% and paradoxical ERK activation accounts for approximately 60–82.4% of the total effects of PLX4720 on tumor growth. **DOI:** http://dx.doi.org/10.7554/eLife.00969.022

While the effects of these BRAFi on JNK-dependent apoptosis is clear and independent of ERK activity, the relative contribution of paradoxical ERK activation vs JNK pathway inhibition to tumorigenesis has not been precisely addressed (Figure 6A). To accomplish this, we took advantage of the fact that paradoxical ERK activation requires intact *CRAF* (Hatzivassiliou et al., 2010; Heidorn et al., 2010; Poulikakos et al., 2010) (Figure 6A). We used isogenic, matched WT and *Craf*-deficient (*Craf*−/−) mouse embryonic fibroblasts (MEFs) and transformed them with adenovirus E1A and human *HRAS*^*G12V*^ to enable anchorage-independent growth (Figure 6—figure supplement 1). These *Craf*−/− cells do not exhibit strong paradoxical MEK or ERK activation, consistent with previous reports (Poulikakos et al., 2010) (Figure 6—figure supplement 1A). Wild-type and matched *Craf*-deficient MEFs were plated in soft agar assays (Su et al., 2012) and treated with PLX4720. Both WT and *Craf*-deficient MEFs exhibited a significant colony formation advantage in the presence of drug (Figure 6B–D). Based upon this analysis, we estimated that the effect of paradoxical ERK activation to be 60% and other effects, including inhibition of JNK activity, to account for the rest (40%) of the total colony growth advantage (Figure 6D). To assess the role of JNK signaling directly, we used *HRAS*^*G12V*^-transformed HaCaT cells with (‘TKD’) and without (‘SCR’) triple lentiviral knockdown of ZAK, MAP4K5, and MKK4 (Figure 3D,E), to perform similar colony formation assays to assess responses to PLX4720 treatment (Figure 6E). Drug treatment conferred a significant colony formation advantage in both sets of cells, which exhibit equivalent paradoxical ERK activation (Figure 6—figure supplement 1B). Yet, untreated TKD HaCaT cells produced more colonies than SCR HaCaT cells suggesting that JNK pathway suppression results in an advantage in the absence of drug and paradoxical ERK activation (Figure 6E). Drug-treated SCR and TKD HaCaT cells, had elevated colony counts to similar levels, as expected, because both lines would experience similar degrees of both paradoxical ERK activation and JNK inhibition, and TKD cells (knocked down for ZAK, MAP4K5, MKK4) are unlikely to experience any further suppression of JNK signaling (Figure 3D). Based on this, we estimated the effect of JNK pathway inhibition to be 17.6% (Figure 6E). When combined with the MEF experiment, we estimate that the effect of JNK inhibition contributes approximately 17.6–40% of the total effect of PLX4720-accelerated colony formation (Figure 6D,E). Importantly, although we can quantify these individual contributions, it is clear in many contexts in cancer that hyperproliferation and inhibition of apoptosis are highly cooperative (Hanahan and Weinberg, 2011), and our data do not preclude the possibility that one or both are individually required.

**Figure 6—figure supplement 1.**
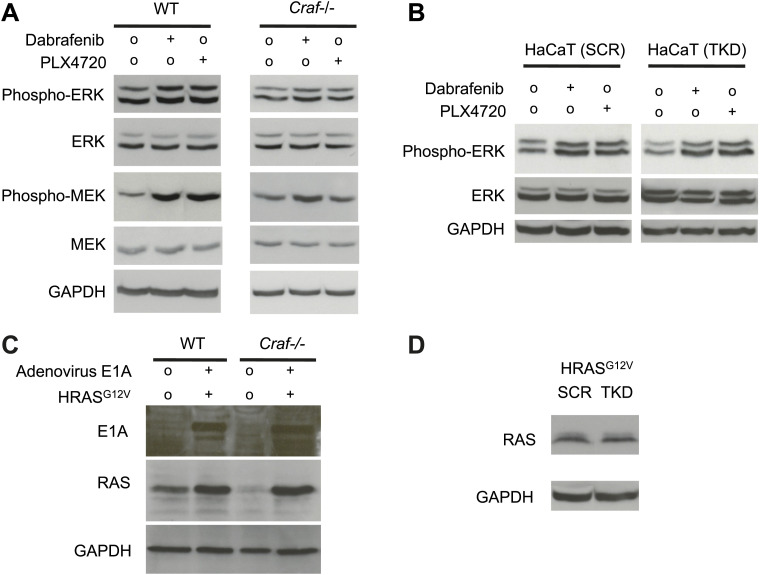
Paradoxical MEK and ERK activation require intact *Craf*. Wild-type (WT) and isogenic *Craf*−/− MEFs were retrovirally transduced with *HRAS*^*G12V*^ and adenovirus E1A thereby enabling anchorage-independent growth for soft agar assays. (**A**) WT MEFs exhibit paradoxical MEK and ERK activation, effects that are significantly reduced in *Craf*−*/*− MEFs, particularly for MEK activation. (**B**) *HRAS*^*G12V*^–transformed HaCaT cells with (‘TKD’) and without (‘SCR’) triple knockdown of ZAK, MAP4K5, and MAP2K4 show equivalent paradoxical ERK activation. (**C**) Transformed WT and *Craf*−/− MEFs show equivalent expression of E1A (sc-25, Santa Cruz) and RAS (sc-32, Santa Cruz). (**D**) Transformed HaCaT cells show equivalent expression of RAS. **DOI:** http://dx.doi.org/10.7554/eLife.00969.023

**Figure 6—figure supplement 2.**
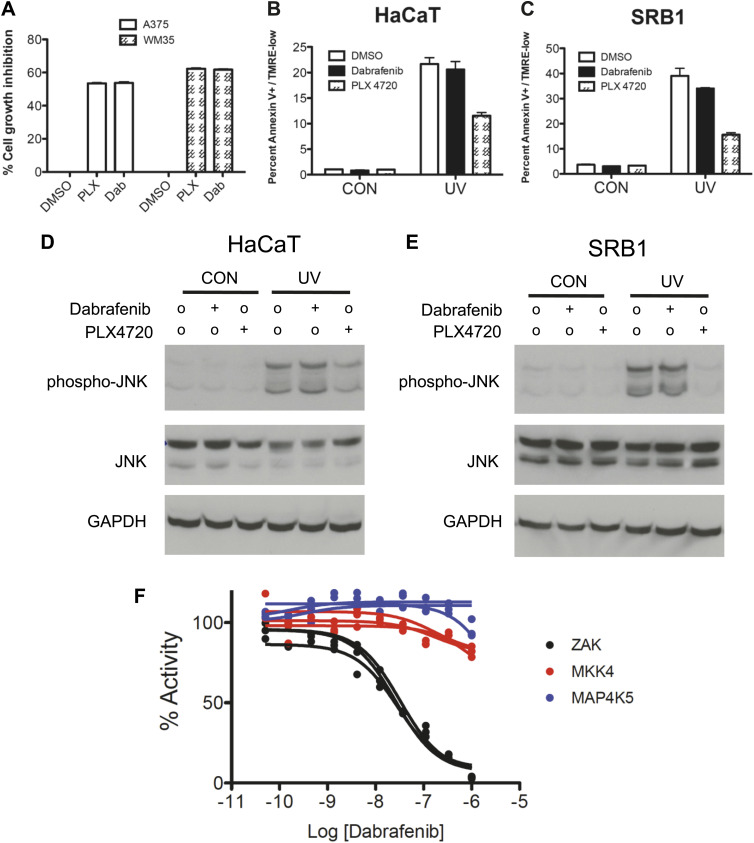
Dabrafenib fails to suppress apoptosis and phospho-JNK upregulation following UV irradiation at bioequivalent doses as compared to PLX4720. Based upon human pharmacokinetic data and in vitro experiments, dabrafenib and PLX4720 were compared in multiple settings at bioequivalent doses (0.05 μM and 1.0 μM, respectively). (**A**) Both BRAFi suppress the growth of A375 and WM35 *BRAF*^*V600E*^ melanoma cell lines to the same degree at these doses. (**B** and **C)** At these doses, dabrafenib fails to suppress UV-induced apoptosis significantly in HaCaT and SRB1 cells. (**D** and **E)** Likewise, dabrafenib fails to suppress phospho-JNK induction, whereas PLX4720 potently suppresses phospho-JNK induction as shown earlier. (**F**) Dabrafenib inhibits ZAK kinase with an estimated IC50 of 28.92 ± 2.23 nM, with no significant inhibition of MAP4K5 or MKK4 up to 1 μM. At 0.01 μM of dabrafenib, the retained activity of ZAK kinase is over 64%. **DOI:** http://dx.doi.org/10.7554/eLife.00969.024

**Figure 6—figure supplement 3.**
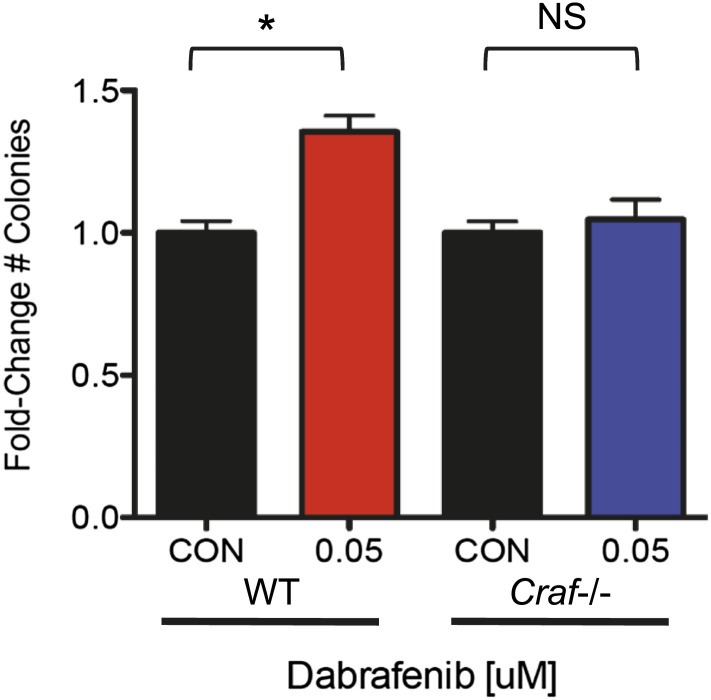
Dabrafenib produces a colony formation advantage only in WT MEFs. At 0.05 μM, dabrafenib produce a significant growth advantage in E1A-*HRAS*^*G12V*^- transformed WT MEFs. In E1A-*HRAS*^*G12V*^-transformed *Craf*−/− MEFs, dabrafenib fails to confer a significant growth advantage, suggesting that in the absence of significant paradoxical ERK activation, dabrafenib does not have a relevant off-target effect that results in a growth advantage. **DOI:** http://dx.doi.org/10.7554/eLife.00969.025

### Dabrafenib and vemurafenib differ significantly in their off-target effects and risk of cSCC

The recently updated combination trial of the BRAFi dabrafenib and MEKi trametinib shows a low 7% cSCC rate in 54 patients, (Flaherty et al., 2012) suggesting that combined MEK inhibition can reduce, but not eliminate cSCC formation, nevertheless reinforcing a role for paradoxical ERK activation (Su et al., 2012). However, clinical trial data on dabrafenib alone at 150 mg PO BID, shows an overall aggregated cSCC rate of 6.1% (Falchook et al., 2012; Hauschild et al., 2012; Long et al., 2012) vs 22% for vemurafenib at 960 mg PO BID in several hundred patients (Flaherty et al., 2010; Chapman et al., 2011; Sosman et al., 2012; Menzies et al., 2013). We interpreted this as being reflective of differences between vemurafenib and dabrafenib, as opposed to unequivocal proof that paradoxical ERK activation is the only mechanism involved. To explore this further, we used similar assays to assess the effects of dabrafenib on apoptosis, JNK signaling, and colony formation. In stark contrast to vemurafenib, dabrafenib has little effect on apoptosis and JNK signaling at doses that are biologically equivalent based upon growth inhibition of *BRAF*^*V600E*^ melanoma cells and human pharmacokinetic data (Falchook et al., 2012; Gowrishankar et al., 2012) (Figure 6—figure supplement 2A). Peak serum concentrations of dabrafenib at 150 mg PO BID in humans (Falchook et al., 2012) are over 50-fold lower (1.55 μM) than mean sustained serum levels of vemurafenib (86 μM) at 960 mg PO BID (Flaherty et al., 2010), and the GI_50_ for the A375 melanoma cell line is less than 0.01 μM for dabrafenib (Greger et al., 2012) vs 0.50 μM for PLX4720 (Tsai et al., 2008). Even at 0.05 μM, dabrafenib did not significantly impact UV-induced phospho-JNK upregulation or apoptosis in HaCaT and SRB1 cells (Figure 6—figure supplement 2B–E). We profiled dabrafenib activity against ZAK, MKK4, and MAP4K5, and found that ZAK is a significant off-target kinase for dabrafenib as well, but at 0.01 μM, over 64% of activity is retained (Figure 6—figure supplement 2F). Neither MKK4 nor MAP4K5 is substantially inhibited by dabrafenib up to 1 μM (Figure 6—figure supplement 2F).

Using transformed WT and *Craf*-deficient MEFs in soft agar assays, we also showed that dabrafenib enhanced colony formation in WT MEFs, but not in *Craf*-deficient MEFs (Figure 6—figure supplement 3). Our results suggest that while both dabrafenib and vemurafenib cause equivalent paradoxical ERK activation in *BRAF*-wild-type cells (Figure 6—figure supplement 1A–B), only vemurafenib confers a significant colony formation advantage in *Craf*-deficient cells that have no significant paradoxical MEK/ERK activation, implicating off-target effects as a key difference between the two drugs with respect to cSCC development (Menzies et al., 2013).

## Discussion

We have discovered an unexpected and novel effect of the BRAFi PLX4720 and vemurafenib in inhibiting apoptosis in vitro and in vivo through the ERK-independent suppression of JNK signaling (Tournier et al., 2000). Our studies implicate the off-target binding and inhibition of these compounds to ZAK primarily (Figure 3—figure supplements 1–3), with additional contributions of MKK4 (MAP2K4) and MAP4K5 inhibition, thus implicating inhibition of JNK signaling at all three upstream tiers of MAP kinase signaling (Figure 3—figure supplement 4). Although MKK4 knockdown alone could suppress UV-induced apoptosis and phospho-JNK induction by up to 27.3% (Figure 3—figure supplement 2), this is expected, given that ZAK signals through MKK4 and MKK7 (Gross et al., 2002) (Figure 2H,I,3E,4H) and MKK4 is important (with MKK7) for full JNK activation (Tournier et al., 2001; Haeusgen et al., 2011). Additionally, UV-mediated induction of NOXA is suppressed in cell lines, primary NHEKs, and in vivo, indicating that this BCL2 family member may be a critical effector of apoptosis in this context (Figures 1G and 4I). In chronically-irradiated Hairless mice, development of well-differentiated papillomas and cSCC is substantially accelerated by PLX4720 treatment without the need for *Ras* mutation and with a dramatic reduction in latency by at least 10 weeks (Figure 5E, Figure 5—figure supplement 1).

While there is enrichment for *RAS* mutations in human cSCC arising in vemurafenib-treated patients vs controls (Oberholzer et al., 2011; Su et al., 2012), up to 30–40% of these lesions do not have *RAS* mutations. Our novel mechanism of BRAFi-mediated apoptosis suppression is the off-target inhibition of several kinases in the JNK pathway, which is independent from, and compatible with, paradoxical ERK–dependent mechanisms (Su et al., 2012) (Figure 6A). Importantly, our approach (Figure 6B–E) has not only allowed us to quantify the contribution of the effect on apoptosis (17.6–40%) vs paradoxical ERK activation (60–82.4%), but also shows that the growth advantage conferred by BRAFi in *BRAF*-WT cells is not accounted for entirely by paradoxical ERK activation. Our results have also shown that there are significant differences between the BRAFi vemurafenib and dabrafenib (Figure 6—figure supplements 2,3) with respect to these off-target effects in cells (even though their relative selectivities for BRAF over ZAK are similar) and this may, in part, explain why they differ in rates of cSCC (Flaherty et al., 2010; Chapman et al., 2011; Falchook et al., 2012; Hauschild et al., 2012; Long et al., 2012; Sosman et al., 2012). At present it is unclear why ZAK appears to be a common off-target kinase and whether structural similarities with other kinases may explain this (Sauter et al., 2010; Wong et al., 2013).

ZAK has been previously studied in the context of bacterial toxin and doxorubicin-mediated cytokine signaling (Jandhyala et al., 2008; Sauter et al., 2010; Stone et al., 2012; Wong et al., 2013), cardiac (Huang et al., 2004) and ischemic stress responses (Su et al., 2012), and in cellular responses to ionizing radiation (Gross et al., 2002; Tosti et al., 2004; Vanan et al., 2012). It is widely expressed across tissues including epidermis, but most prominently in heart, liver, and muscle (Abe et al., 1995; Miyata et al., 1999; Liu et al., 2000; Bloem et al., 2001; Gross et al., 2002; Su et al., 2004), and has purported tumor suppressive roles in lung cancer (Yang et al., 2010) and tumor promoting ones in partially transformed mouse skin epidermal cells (Cho et al., 2004). ZAK is a MAP3K that is upstream of both JNK and p38 signaling (Gross et al., 2002; Tosti et al., 2004; Jandhyala et al., 2008; Cheng et al., 2009; Stone et al., 2012; Wong et al., 2013) and signals to JNK through MKK4 and MKK7 (Gross et al., 2002) (Figures 2H,I,3E,4H). Accordingly, macrophages derived from ZAK-deficient mice have profound defects in activation of both JNK and p38 signaling following doxorubicin exposure (Wong et al., 2013). In the setting of UV-induced apoptosis as we have examined here, JNK activity is the major driver of apoptosis (Derijard et al., 1994; Chen et al., 1996; Tournier et al., 2000), also by virtue of the fact that phospho-p38 induction by UV is inconstant (Figures 1F,H,2B,4H); although where it is induced, PLX4720/vemurafenib treatment suppresses it (Figure 1F (SRB1, HaCaT cells), Figures 1H,2B). These results are consistent with the model (Figure 3—figure supplement 4) that ZAK signals to both JNK and p38, but is principally necessary for activating JNK in stress-induced apoptosis.

Because off-target kinases in the JNK pathway are affected by vemurafenib/PLX4720, one expects that these kinases would be affected in all cells regardless of *BRAF* status. Indeed, melanoma cells expressing BRAF^V600E^ also exhibit suppression of JNK activity following irradiation (Figure 1H). However, in *BRAF*^*V600E*^-expressing melanoma cells, the effect of blocking BRAF activity alone clearly dominates, because these cells are exquisitely dependent upon BRAF activity (Tsai et al., 2008). Therefore, although off-target kinases are inhibited, the cellular context of dependence on particular kinases is still highly relevant and likely dictates the outcome.

Our findings suggest a tumor suppressive role for JNK signaling in the context of drug-induced cSCC, though the role of JNK in cancer is highly context-dependent and is partly related to differing functions of the individual isoforms and partial redundancy (Tournier et al., 2000). Nonetheless, there is ample in vivo evidence showing that JNK can function in a tumor suppressive role. Genetically-engineered mice lacking *Jnk1* and *Jnk2* have increased (She et al., 2002) and decreased (Chen et al., 2001) susceptibility, respectively, to chemical carcinogenesis in skin, though these mice also have opposite defects in epidermal differentiation (Weston et al., 2004). In mouse models, lack of *Jnk1/2* activity suppresses *Ras*-driven tumorigenesis in lung (Cellurale et al., 2011) and promotes it in *Ras*-driven and *Trp53*-deficient breast cancer models (Cellurale et al., 2010, 2012). In the context of *Pten*-deficiency, loss of *Jnk1/2* or *Mkk4*/*Mkk7* promotes aggressive prostate adenocarcinoma (Hubner et al., 2012). Importantly, the effects of JNK on cancer are not always tumor cell autonomous, as JNK activity supports a pro-tumorigenic inflammatory microenvironment in hepatocellular carcinoma (Das et al., 2011).

Our results have important clinical implications and suggest careful consideration of combining certain BRAFi with therapeutic modalities that induce apoptosis such as radiation or chemotherapy, particularly with respect to off-target tissues (keratinocytes in skin). We have shown that off-target inhibition of kinases, even at higher IC50s, can contribute biologically significant effects, particularly if they are in the same pathway. Finally, our results show that kinase inhibitors must be considered in terms of their entire spectrum of activity, which can dramatically affect pathways distinct from those affected by inhibition of the intended target.

## Materials and methods

### Ethics statement

All studies were conducted under institutionally-approved IRB (LAB08-0750) and ACUF (06-09-06332) protocols for the protection of human and animal subjects, respectively.

### Cell lines

Cutaneous SCC cell lines (SRB1, SRB12, COLO16) were obtained from Jeffrey N Myers (MD Anderson), HaCaT cells from Norbert Fusenig (German Cancer Research Center), and WM35 and A375 melanoma cell lines from Michael Davies (MD Anderson). The cell lines were validated by STR DNA fingerprinting using the AmpFℓSTR Identifiler kit according to manufacturer instructions (Applied Biosystems, Grand Island, NY). The STR profiles were compared to known ATCC fingerprints (ATCC.org), to the Cell Line Integrated Molecular Authentication database (CLIMA) version 0.1.200808 (http://bioinformatics.istge.it/clima/) and to the MD Anderson fingerprint database. The STR profiles matched known DNA fingerprints (HaCaT) or were unique (SRB1, SRB12, COLO16). The cells were cultured in DMEM/Ham’s F12 50/50 (Cellgro) supplemented with 10% Fetal Bovine Serum (FBS) (Sigma), glutamine, and Primocin (Invivogen). NHEKs (Lonza) were cultured in media according to manufacturer’s instructions. Irradiation was performed using an FS40 sunlamp dosed by an IL1700 radiometer. Following irradiation, cells were treated with PLX4720 (Plexxikon), vemurafenib (Selleck Chemicals) or DMSO (1:2000).

### Antibodies

Primary antibodies (Cell Signaling) used for Western blot analysis included p53 (2527P, clone 7F5), phospho-/total p44/42 MAPK (4370S, cloneD13.14.4E/9102S), phospho-/total p38 MAPK (4511S, clone D3F9/9212S), phospho-/total JNK (4668S, clone 81E11/9252S), BIM (2933, clone C34C5), MCL1 (5453P, clone D35A5), cleaved caspase-3 (9661L, clone D175), phospho-/total MKK7 (4171S/4172S), phospho-/total MKK4 (9156S/9152S), phospho-/total MEK (9121S/9122), MAP4K5 (ab56848; Abcam) and NOXA (mA1-41000; Thermo Scientific). GAPDH (21,182, clone 14C10; Cell Signaling) and beta-actin (A5060; Sigma) were probed to ensure even loading of protein samples. Immunohistochemistry was performed for phospho-JNK (V7931; Promega) and cleaved caspase-3 (Cell Signaling as above). Antibody against ZAK was generously provided by R Ruggieri (Feinstein Institute for Medical Research).

### Flow Cytometry

TMRE (Invitrogen) was used as a measure of mitochondrial membrane potential, Annexin V-FITC or Annexin V-APC (Invitrogen) as a probe for apoptosis, and Sytox Blue (Invitrogen) as an indicator for dead cells. At 24 hr post-irradiation, floating and adherent cells were collected and stained with TMRE, Annexin V and Sytox Blue. Data was collected and analyzed using a flow cytometer (Fortessa, Becton Dickinson) and FlowJo Software (Tree Star). Data were calculated and charts were plotted using GraphPad Prism 5 software.

### Western blot analysis

Cell were lysed in standard buffers with protease inhibitors (Roche) and phosphatase inhibitors (Santa Cruz) with extracts run on SDS/polyacrylamide gels and transferred to Immobilon-P transfer membrane (Millipore). Blots were blocked in TBST (10 mM Tris-HCL pH8, 150 mM NaCl, 0.5% Tween) with milk or BSA, probed with primary antibodies, corresponding HRP-conjugated secondary antibodies, and signals detected using ECL kit (Amersham).

### Immunohistochemistry and histology

Cutaneous squamous cell carcinomas biopsied from patients treated with or without BRAF-inhibitor were obtained either under clinical trials (Roche) or separate IRB approval (LAB08-0750). Staining levels were quantified by counting positively labeled cells and dividing by the total area of the tumor tissue within each sample. To measure tumor areas, all samples were photographed, tumor cells outlined, and total pixel numbers calculated using included image analysis tools in Adobe Photoshop and standardized to a hemacytometer to convert to mm^2^. To measure apoptosis in irradiated skin, pyknotic or dyskeratotic epidermal keratinocytes were counted and normalized to length (mm) of epidermis.

### Kinase activity profiling

PLX4720 and vemurafenib were prepared in DMSO and tested in duplicate at four concentrations (50 nM, 200 nM, 1000 nM, 10 μM) against a panel of 38 kinases using a quantitative competitive binding assay (KINOMEscan, San Diego, CA). Average percent inhibition was reported. Estimated *K*_*d*_ values were derived by averaging pointwise estimates calculated using a transformed Hill equation at each concentration of drug. In vitro kinase assays were performed using human full-length ZAK (MBP substrate, ATP 2.5 μM), amino acids 33-end MKK4 (JNK1 substrate, ATP 0.1 μM), and full length MAP4K5 (MBP substrate, ATP 10 μM) (Reaction Biology). Assays for BRAF^V600E^ and ASK1 against vemurafenib were run in parallel revealing IC50s of 31.6 ± 2.9 nM for BRAF^V600E^ and no significant inhibition of ASK1, as previously reported (Bollag et al., 2010).

### Lentiviral knockdown experiments

Lentiviral shRNA knockdown was accomplished using standard lentiviral methods using 293T cells and psPAX2/pVSV.G packaging plasmids. shRNA clones against ZAK (clones V2LHS_239842, V3LHS_336769), MKK4 (clones V3LHS_646205, V3LHS_386825A), and MAP4K5 (clones 196277A, 334084), as well as a non-silencing shRNA were obtained from Open Biosystems in the GIPZ vector. Following transduction, cells were puromycin-selected and FACS sorted to obtain cells with high-level suppression. Degree of mRNA suppression was quantified by qPCR using Taqman probes using internally controlled (2-color, same well) GAPDH probes to ensure proper normalization.

### ZAK overexpression

ZAK (T82Q) mutant was generated in the pcDNA3 mammalian expression vector. HaCaT cells were electroporated using the Neon transfection system 24 hr prior to irradiation. Transfection efficiencies were estimated to be 70–80% by GFP fluorescence.

### Mouse experiments

Wild-type C57BL/6 mice, 5–8 weeks old, were pretreated with PLX4720 in 5% DMSO in 1% methylcellulose for 2–4 days at 40–80 mg/kg twice a day or control 5% DMSO in 1% methylcellulose by oral gavage. The mice were shaved and depilated (Nair) 24 hr prior to irradiation with a solar simulator (Oriel) dosed at 10 kJ/m^2^ of UVB. Epidermis was harvested and protein extracts run on Western blots and probed as above. For chronically-irradiated Hairless mice, 3–4 week old males were irradiated thrice weekly for a total weekly dose of 12.5 kJ/m^2^ UVB (solar simulator, Oriel). At 72 days, PLX4720 treatment was started using drug-impregnated chow (Plexxikon) with vehicle chow in the control cohort.

### Soft agar assays

Following plating of bottom agar (0.6% Bacto Agar) with media and appropriate amounts of drug, 2500 to 10,000 cells per well (transformed WT, *Craf*−/− MEFs; transformed HaCaT SCR and TKD cells) were embedded in top agar (0.3%) and plated in 24-well plates. Control or drug-treated media was replaced every 48 hr for 4–6 weeks. The plates were stained with 1% crystal violet and colonies counted by bright-field microscopy.

### Statistical analysis

All data are represented as means ± SEM. All experiments were performed in triplicate at least. Student’s t-test was used for comparison between two groups. p≤0.05 was considered significant.
