# Correction: BRAF inhibitors suppress apoptosis through off-target inhibition of JNK signaling

**DOI:** 10.7554/eLife.111028

**Published:** 2026-02-19

**Authors:** Harina Vin, Sandra S Ojeda, Grace Ching, Marco L Leung, Vida Chitsazzadeh, David W Dwyer, Charles H Adelmann, Monica Restrepo, Kristen N Richards, Larissa R Stewart, Lili Du, Scarlett B Ferguson, Deepavali Chakravarti, Karin Ehrenreiter, Manuela Baccarini, Rosamaria Ruggieri, Jonathan L Curry, Kevin B Kim, Ana M Ciurea, Madeleine Duvic, Victor G Prieto, Stephen E Ullrich, Kevin N Dalby, Elsa R Flores, Kenneth Y Tsai

**Keywords:** Human, Mouse

Vin H, Ojeda SS, Ching G, Leung ML, Chitsazzadeh V, Dwyer DW, Adelmann CH, Restrepo M, Richards KN, Stewart LR, Du L, Ferguson SB, Chakravarti D, Ehrenreiter K, Baccarini M, Ruggieri R, Curry JL, Kim KB, Ciurea AM, Duvic M, Prieto VG, Ullrich SE, Dalby KN, Flores ER, Tsai KY. 2013. BRAF inhibitors suppress apoptosis through off-target inhibition of JNK signaling. *eLife* **2**:e00969. doi: 10.7554/eLife.00969.

Published 5 November 2013

This correction is published to address concerns originally posted anonymously to PubPeer.

The main point of Figure 1F was to show that UV induced phospho-JNK activation is suppressed by the BRAF inhibitor (PLX4720). The point of including pERK expression was to ensure that the drug was inducing expected changes, notably paradoxical ERK activation.

In reviewing the original data, we have come to realize that the data for phospho-ERK (“pERK”) in that row (for the three cell lines SRB1, SRB12, COLO16) were indeed spliced in, and the published figure was a result of several blots that were inadvertently and unintentionally blended and submitted as the published composite figure. No revision to the text is made. The corrected Figure 1 (updated for panel F, pERK SRB1 SRB12 COLO16 bands) is shown here:

**Figure 1:**
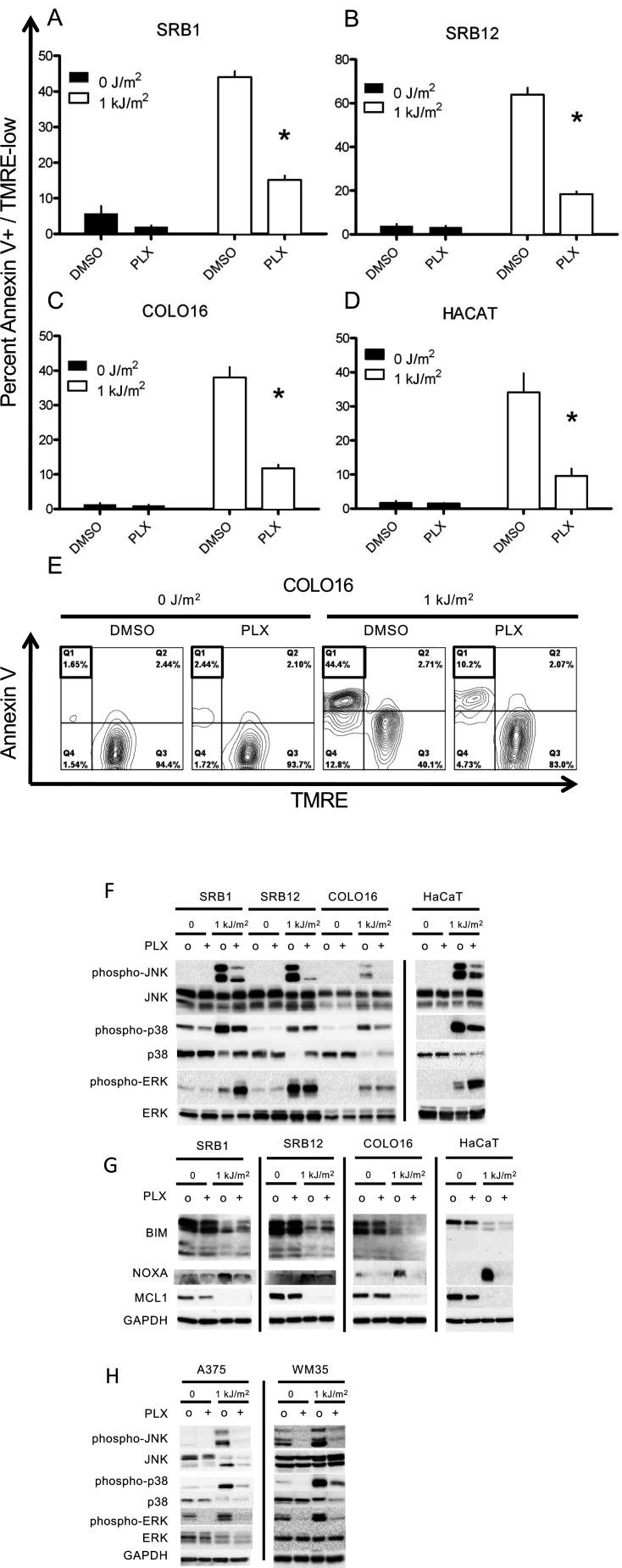


The originally published Figure 1 is shown here for reference.

**Figure 2:**
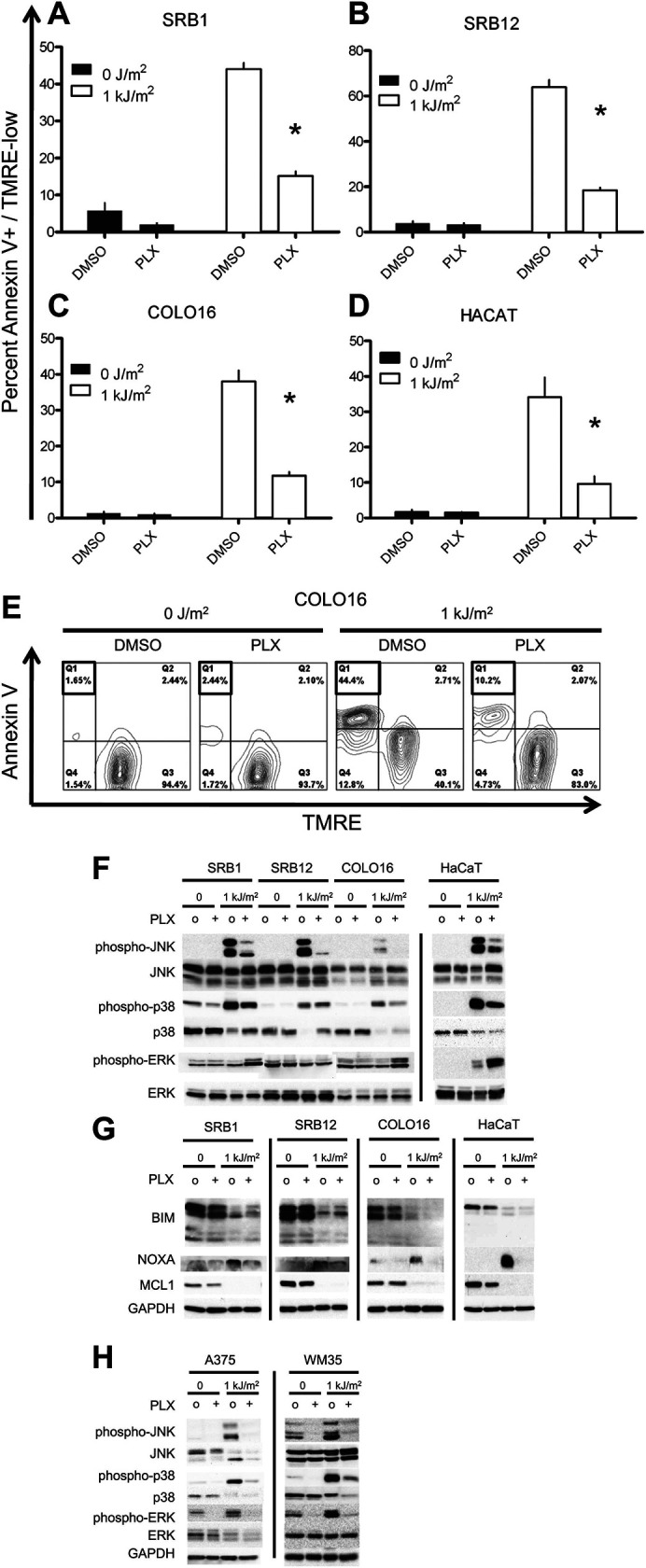


The Figure 1F legend has been updated:

(**F**) Western blots probed for the MAP kinases demonstrated strong phospho-JNK and phospho-p38 induction following irradiation and significant suppression by PLX4720. Phospho-ERK was induced following irradiation, and at 24 hr, paradoxical hyperactivation in the presence of PLX4720 was observed in SRB1 and HaCaT cells.

The originally published Figure 1F legend is shown. Underline indicates differences with the updated text:

Western blots probed for the MAP kinases demonstrated strong phospho-JNK and phospho-p38 induction following irradiation and significant suppression by PLX4720. Phospho-ERK was slightly induced following irradiation, and at 24 hr, paradoxical hyperactivation in the presence of PLX4720 was observed, particularly in SRB1 and HaCaT cells.

The article has been corrected accordingly. Original blots related to Figure 3E were additionally reviewed by the Editorial staff at eLife and accurately reflect the presented data. The scientific merit of the conclusions remains supported by the data.

